# Tubulin tyrosine ligase variant perturbs microtubule tyrosination, causing hypertrophy in patient-specific and CRISPR gene-edited iPSC-cardiomyocytes

**DOI:** 10.1172/jci.insight.187942

**Published:** 2025-08-08

**Authors:** Pratul Kumar Jain, Susobhan Mahanty, Harshil Chittora, Veronique Henriot, Carsten Janke, Minhajuddin Sirajuddin, Perundurai S. Dhandapany

**Affiliations:** 1 Cardiovascular Development and Disease Mechanisms, Institute for Stem Cell Science and Regenerative Medicine, (inStem), Bangalore, India.; 2 The University of Trans-Disciplinary Health Sciences and Technology, Bangalore, Karnataka, India.; 3 National Centre for Biological Sciences, Tata Institute of Fundamental Research, GKVK Campus, Bangalore, India.; 4 Institut Curie, PSL Research University, CNRS UMR3348, Orsay, France.; 5 Université Paris-Saclay, CNRS UMR3348, Orsay, France.

**Keywords:** Cardiology, Genetics, Stem cells, Heart failure, Human stem cells

## Abstract

Hypertrophic cardiomyopathy (HCM) is a hereditary heart condition characterized by either preserved or reduced ejection fraction without any underlying secondary causes. The primary cause of HCM is sarcomeric gene mutations, which account for only 40%–50% of the total cases. Here, we identified a pathogenic missense variant in tubulin tyrosine ligase (TTL p.G219S) in a patient with HCM. We used clinical, genetics, computational, and protein biochemistry approaches, as well as patient-specific and CRISPR gene-edited induced pluripotent stem cell–derived cardiomyocytes (iPSC-CMs), to demonstrate that the TTL pathogenic variant results in a reduced enzymatic activity and the accumulation of detyrosinated tubulin leading to the disruption of redox signaling, ultimately leading to HCM. Our findings highlight — for the first time to our knowledge — the crucial roles of the TTL variant in cardiac remodeling resulting in disease.

## Abstract 2

Tubulin tyrosine ligase variant perturbs microtubule tyrosination causing hypertrophy in patient-specific and CRISPR gene-edited iPSC-cardiomyocytes

## Introduction

Hypertrophic cardiomyopathy (HCM) is a cardiac muscle disease that affects approximately 1 in every 250–500 individuals (1–3). Patients with HCM have an increased risk of heart failure and sudden cardiac death (1–3). This condition is typically characterized by an enlargement of the heart muscle, with either preserved or increased ejection fraction (EF) and contractility defects in the absence of other cardiac, systemic, or metabolic diseases that can independently cause left ventricular hypertrophy. The majority of patients with HCM are represented by gene variants in sarcomeric protein genes (1–4). Strikingly, 30%–40% of patients with HCM do not exhibit mutations in sarcomeric genes (1–4).

Microtubules (MTs) have recently been discovered to play an important mechanotransduction role during the heart contraction. They contribute to the stability and organization of the actin cytoskeleton and influence the intracellular transport and distribution of essential proteins and organelles, thereby affecting cardiac function and response to mechanical stress (5). The detyrosination/tyrosination cycle is a mechanism known to regulate MT-related mechanics in cardiomyocytes (CMs) (5, 6). The posttranslational modification of α-tubulin by the detyrosination/tyrosination cycle occurs through alternating removal and addition of tyrosine at its carboxyl terminus. This process, which is mediated by detyrosinating enzymes and the tubulin tyrosine ligase (TTL) (6, 7). The level of tubulin tyrosination governs the stability and dynamics of MTs and is required for redox balance (7, 8). However, to date, no human-specific *TTL* variant has been reported as a cause for HCM.

In this study, we identified a heterozygous variant in the *TTL* gene, where glycine is substituted by serine at residue 219 (p.G219S), in a South Indian patient with HCM. This mutation results in reduced TTL activity, leading to the accumulation of detyrosinated MTs and oxidative stress in patient-specific and CRISPR gene-edited iPSC-derived CMs, resulting in cardiac hypertrophy.

## Results

### Identification of TTL as a HCM gene.

**Figure 1:**
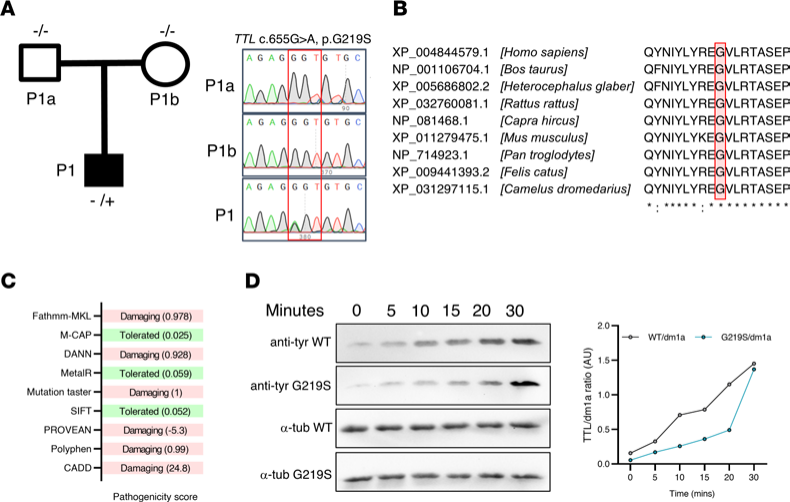
Identification of TTL variant and enzyme activity assay. (**A**) Pedigree of the HCM family with the TTL p.G219S variant. Black shaded boxes represent the affected individuals; (+) and (–) represent the presence and absence of variant in the individual, respectively, and (–/+) represents the presence of heterozygous condition with respect to the variant. On the right is the Sanger sequencing analysis for the P1 and his family members. (**B**) Multiple sequence alignment of the protein region across different species with amino acid conservation at site 219 is shown in the red box. (**C**) In silico analysis of the *TTL* gene variant using various computational tools. (**D**) Tyrosination assay with purified TTL WT and TTL p.G219S proteins.

We exome sequenced and analyzed a cohort of 101 patients with HCM that were negative for known HCM genes (9). The details of the patient clinical characteristic features and exome sequencing pipeline were reported in our previous publication (9). Among the 101 patients with HCM analyzed, we identified a gene variant (c.655G>A) in the coding sequence of *TTL* in a patient referred to as patient 1 (P1). The patient’s healthy family members, P1a and P1b, tested negative for the specific variant, suggesting a de novo origin in the individual with the *TTL* variant (Figure 1A). In the proband, the glycine residue at position 219 was replaced by a serine residue (p.G219S) in TTL. This position is highly conserved across the species (Figure 1B), and various in silico tools have predicted a *TTL* variant to be damaging or deleterious in nature (Figure 1C). The variant was observed as an extremely ultrarare variant in Genome Aggregation Consortium (gnomAD) (4 of 1,224,800 European and East Asian alleles; minor allele frequency [MAF] = 3.27 × 10^–6^), but was absent in South Asian ancestry (0 of 91,036 alleles), and Genotype to Mendelian Phenotype (Geno2MP v2.6) cohort (0 of 38,688 alleles) belonging to various families with different spectrum of diseases. Also, it was absent in data sets from Indian and White Wellderly controls, including healthy aging Indian exomes (www.instem.res.in/IndiCardiome) and the Wellderly data set, as well as the South Asia-specific Indian control data (*N* = 3521 alleles) sets such as Genome Asia 100K, Indigenomes, and South Asian (Indian) healthy controls.

### TTL p.G219S patient clinical characteristics.

Genotype-phenotype correlations revealed that P1 was diagnosed with HCM at the age of 40 (onset age). The patient had left ventricular internal diameter end diastole (LVIDd) and left ventricular internal diameter end systole (LVIDs) measurements of 46 and 23 mm, respectively, with an left ventricular ejection fraction (LVEF) of 70% and an IVS of 23 mm. The proband showed the absence of any other secondary HCM causes such as hypertension or diabetes and was treated with β-adrenergic blockers.

### TTL p. G219S showed perturbed conformational dynamics compared with the WT.

To further investigate the effect of the mutation in the TTL protein on its interaction with the α-tubulin carboxy terminal tail (CTT), we performed molecular dynamics (MD) simulation of the WT and mutant TTL enzyme with CTT using GROMACS (10). TTL-bound α-tubulin CTT structure was obtained from the crystal structure of the α-tubulin-stathmin-TTL ADP complex (16) (PDB ID: 4IHJ) by removing the stathmin-bound α-tubulin dimers using UCSF Chimera (11). A single amino acid substitution at the glycine 219 position to serine was introduced in the WT protein using UCSF Chimera to generate the mutated version of the protein (Supplemental Figure 1A; supplemental material available online with this article; https://doi.org/10.1172/jci.insight.187942DS1). The deviation in the structural characteristics, spatial displacement, and stability of the protein was monitored during the course of the 100 ns simulation and was represented using root-mean square deviation (RMSD), root mean square fluctuation (RMSF), and change in the radius of gyration (Rg) (Supplemental Figure 1, B–D). The RMSD fluctuation of the α carbons (Cα) backbone shown in Supplemental figure 1B indicated that, even though fluctuations were evident throughout the simulation, the WT system reached its equilibrium after approximately 8 ns with the structure maintaining at a level of around 1.5 Å until the end of the simulation, whereas the mutant TTL system reached equilibrium after 20 ns and showed a higher average displacement among atoms, with structures maintained above 2 Å. The change in the RMSF was used to identify protein regions with greater flexibility. In comparison with the WT, the mutant exhibited higher flexibility in residues near site 219, which are in close proximity to the highly conserved nucleotide binding site (7). The measure of Rg reflects the overall compactness of the protein that correlates with the stability of the protein (12). The overall changes in the Rg value of the WT and mutant during the course of simulation are shown in Supplemental Figure 1D. Our results suggest that the mutant protein has a higher degree of Rg than the WT protein, indicating a reduction in compactness in the mutant TTL variant.

### TTL p.G219S protein exhibited delayed enzymatic activity.

MD simulation suggested that the variant site of TTL p.G219S induced a conformational change near the β loop and the nucleotide binding site and altered its interacting ability with the α-tubulin CTT. To validate this observation, we conducted an in vitro tyrosination assay using purified recombinant WT and mutant TTL proteins (Figure 1D) with carboxypeptidase-treated α-tubulin as the substrate. The enzymatic activities of both WT and mutant TTL were determined by immunoblotting at different time points. Our findings reveal that the rate of tyrosine addition to the α-tubulin substrate by the mutant TTL protein was notably delayed compared with that of the WT TTL protein (Figure 1D).

### TTL p.G219S patient-specific iPSC generation and characterization.

To understand the molecular mechanisms, we generated an induced pluripotent stem cell (iPSC) line from the P1 carrying the TTL p.G219S variant using the Sendai reprogramming method. In brief, the patient iPSC line displayed embryonic stem cell (ESC) morphology, expressed pluripotency markers, differentiated into 3 germ layers, and exhibited normal karyotype (Supplemental Figure 2, A–G).

### TTL p.G219S patient-specific iPSC-CMs display HCM phenotypes.

**Figure 2:**
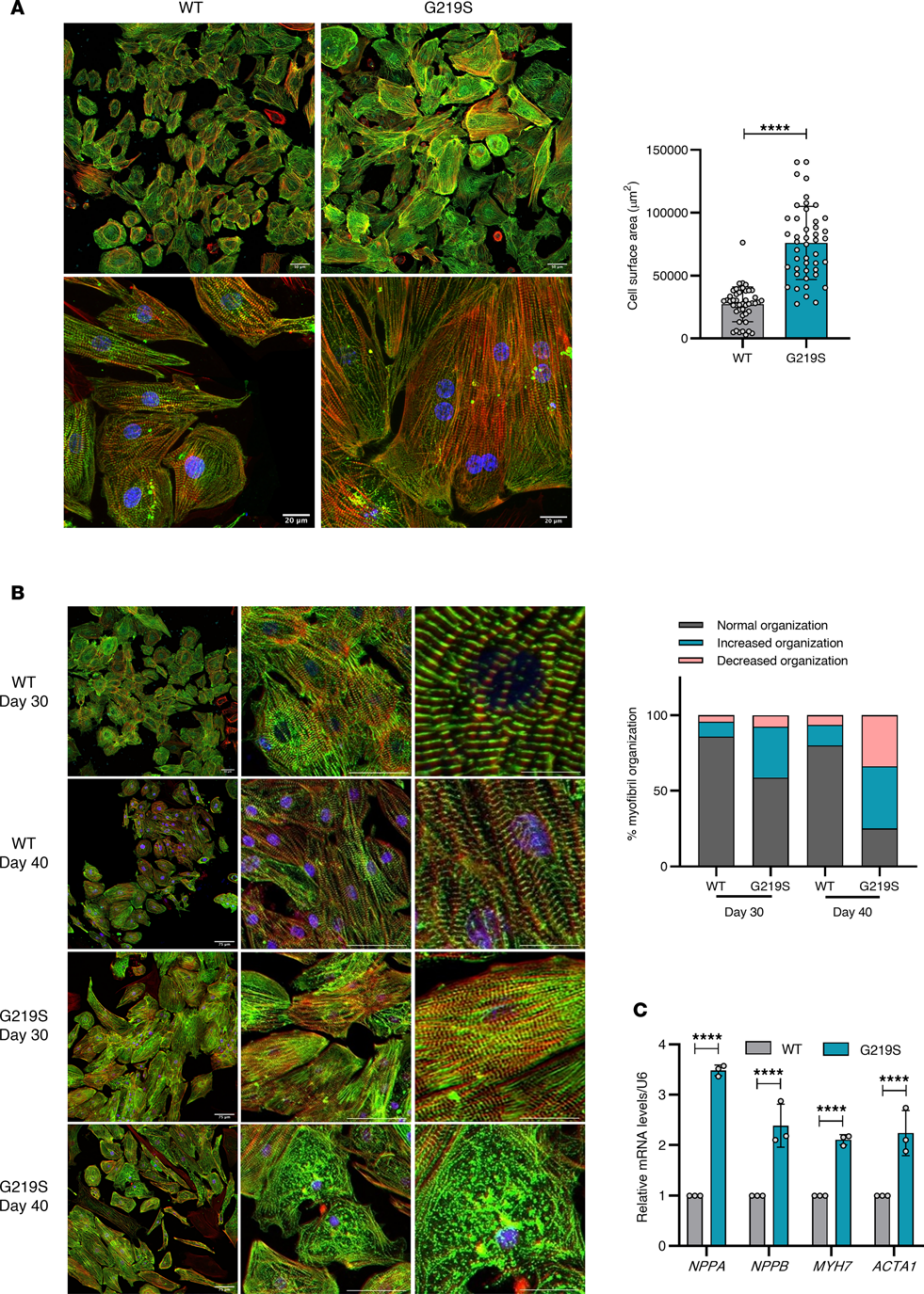
TTL p.G219S iPSC-CMs exhibit hypertrophic phenotypes. (**A**) Representative images of iPSC-CMs stained for α-sarcomeric actinin (green), phalloidin (red), and Hoechst (blue). (**B**) Quantification of the percentage of cells with increased organization and decreased disorganization at days 30 and 40 of differentiation. Values are shown as mean ± SEM with each experiment performed in triplicate (*n* = 60–90 cells per group). (**C**) qPCR analysis of the hypertrophic markers (*NPPA*, *NPPB*, *MYH7,* and *ACTA1*). The mRNA levels were normalized to those of U6. Significance was evaluated by Student’s *t* test or multiple *t* test. ****P < 0.0001. Scale bars: 75 μm (above), 20 μm (below) (**A**), 75 μm (**B**).

To elucidate cardiac pathophysiology, TTL p.G219S iPSC lines were differentiated into CMs (iPSC-CMs) by modulating the Wnt/β-catenin pathway (13), followed by lactate-based selection and purification, and stained for sarcomere protein (α-sarcomeric actinin [ACTN2]) (Figure 2A). TTL p.G219S iPSC-CMs showed a marked increase in cell surface area in comparison to its WT (Figure 2A).

The myofibrillar system is crucial for cardiac contraction and undergoes significant intrinsic changes during the development of HCM (14, 15). To study myofibrillar organization and sarcomeric integrity, we utilized ACTN2 immunofluorescence staining as a marker. Our findings reveal a substantial increase in sarcomeric organization at day 30 and shifting to a subsequent increase in sarcomeric disorganization at day 40 in CMs derived from TTL (p.G219S) compared with WT (Figure 2B). Additionally, the relative mRNA levels of bona fide hypertrophy markers like atrial natriuretic peptide (*NPPA*), brain natriuretic peptide (*NPPB*), actin α 1 (skeletal muscle) (*ACTA1*), and β myosin heavy chain (*MYH7)* ratio were significantly upregulated in TTL p.G219S iPSC-CMs (Figure 2C).

**Figure 3:**
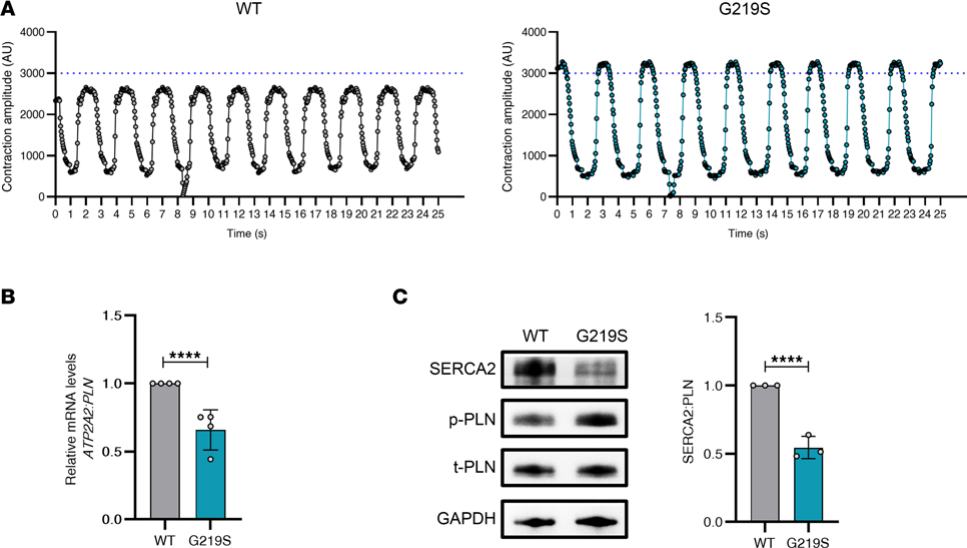
TTL p.G219S iPSC-CMs display contractile defects. (**A**) Comparison of iPSC-CM contractile physiology with respect to time using the muscle motion plug-in in ImageJ (NIH). (**B**) qPCR analysis of *ATP2A:PLN*, involved in calcium regulation in sarcomeres. (**C**) Representative immunoblots of SERCA2a and PLN levels in iPSC-CMs and their quantification on the right side. (Phosphorylation site for PLN is Ser16/17). Values are shown as mean ± SEM with each experiment performed in triplicate. Significance was evaluated by Student’s *t* test. ****P < 0.0001.

Furthermore, mutant CMs exhibited reduced expression of sarcoplasmic reticulum calcium ATPase (SERCA2a)/*ATP2A2* and increased phosphorylation of phospholamban (PLN), indicating a reduction in the reuptake of cytosolic Ca^2+^ into the sarcoplasmic reticulum and abnormal contractility due to increased amplitude possibly leading to reduced kinetics of contraction (Figure 3A). Our results show considerably lower expression of SERCA2a in response to PLN levels (Figure 3, B and C). Notably, mutant CMs exhibited a greater magnitude of contraction than WT CMs, suggesting that the increased contractile amplitude is a result of hypertrophy-induced contraction force. In summary, upregulation of hypertrophy markers, increase in cell size, sarcomeric disarray, and contractile dysfunction through abnormal calcium-handling proteins suggest that the TTL (p.G219S) variant can lead to pathological cardiac hypertrophy.

### TTL p.G219S iPSC-CMs displayed increased detyrosinated α-tubulin levels.

**Figure 4:**
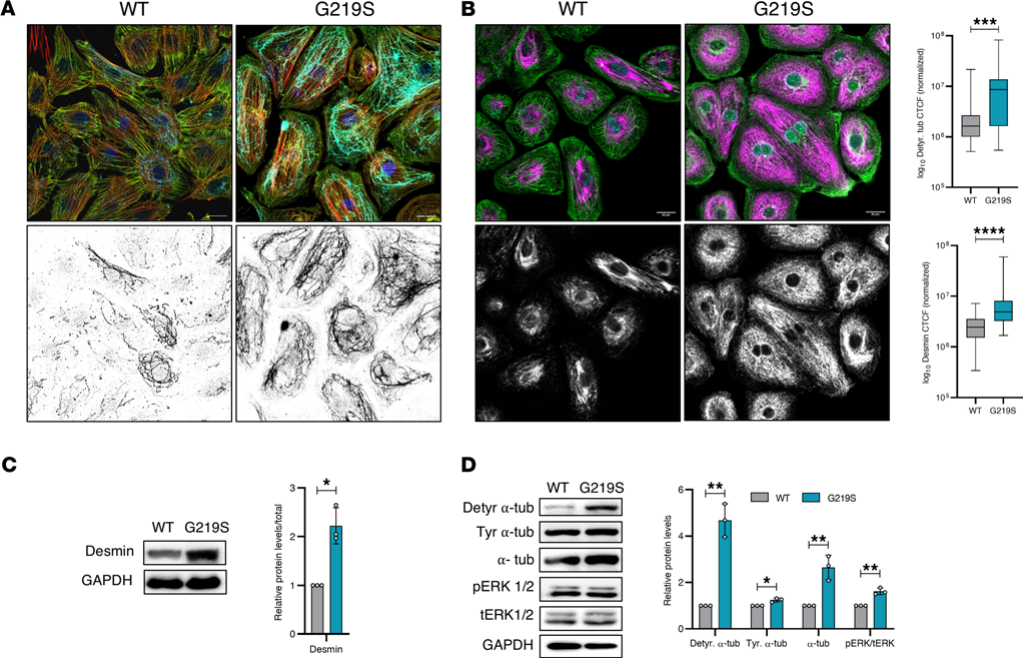
TTL p.G219S iPSCs-CMs showing increased levels of detyrosinated α-tubulin and desmin. (**A**) Representative immunofluorescence images of detyrosinated α-tubulin (cyan), α-sarcomeric actinin (green), phalloidin (red), and Hoechst (blue) in WT and TTL p.G219S iPSC-derived cardiomyocytes. Lower panel is the grayscale of the detyrosinated α-tubulin form of the same image of the respective upper panel. (**B**) Immunofluorescence images of desmin (magenta), α-sarcomeric actinin (green), and Hoechst (cyan) in WT and TTL p.G219S iPSCs-CMs. Lower panel is the grayscale of the desmin of the same image of the respective upper panel. Levels of the detyrosinated α-tubulin form and desmin were quantified based on intensity, and corrected total cell fluorescence was calculated, as shown on the right side (*n* = 50–60 cells per group). Both the quantification (**A** and **B**) are provided on the right of **B**. (**C**) Immunoblots for desmin from the lysates from WT and TTL p.G219S iPSCs-CMs, with quantification on the right. (**D**) Representative immunoblots from total lysates of WT and TTL p.G219S iPSCs-CMs, as quantified on the right. Values are shown as mean ± SEM with each experiment performed in triplicate. Significance was evaluated by Student’s *t* test or multiple *t* test. *P < 0.05, **P < 0.01, ***P < 0.001, and ****P < 0.0001. Scale bar: 20 μm.

**Figure 5:**
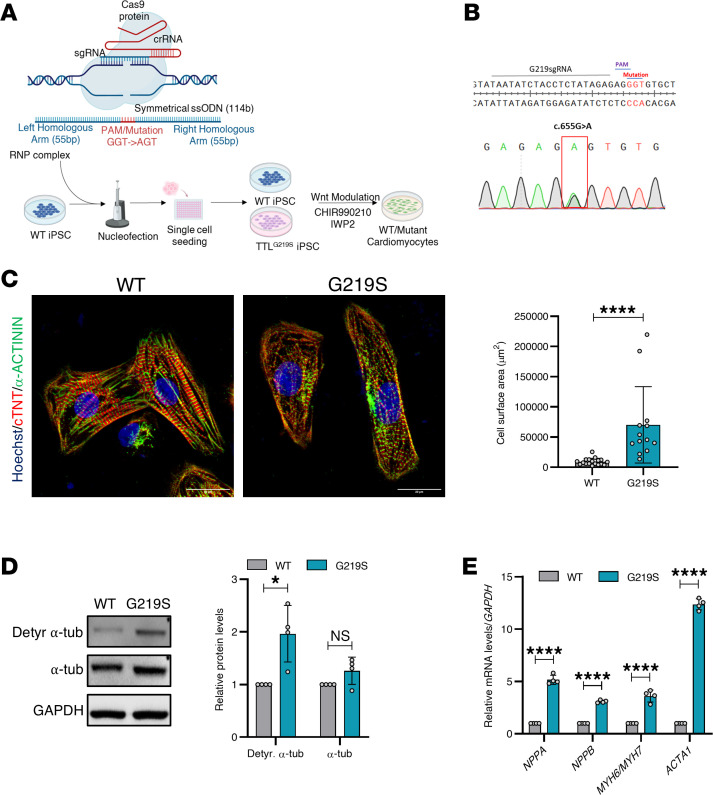
TTL p.G219S gene-edited isogenic iPSCs-CMs showing increased cell size, tubulin detyrosination, and fetal gene reexpression. (**A** and **B**) Schematic figure illustrating the CRISPR/Cas9 gene editing strategies in WT iPSC background to generate isogenic TTL p.G219S and Sanger sequencing confirmation of the respective variant. (**C**) Representative images of iPSC-CMs stained for α-sarcomeric actinin (green), phalloidin (red), and DAPI (blue). (**D**) Immunoblots from the lysates from WT and TTL p.G219S iPSCs-CMs, with quantification on the right side. (**E**) qPCR analysis of the hypertrophic markers (*NPPA*, *NPPB*, *MYH7,* and *ACTA1*). The mRNA levels were normalized to those of U6. Values are shown as mean ± SEM with each experiment performed in triplicate. Significance was evaluated by Student’s *t* test or multiple *t* test. *P < 0.05, and ****P < 0.0001. Scale bar: 20 μm.

TTL plays an important role in the tyrosination/detyrosination process of MTs (7, 16, 17). Specifically, detyrosinated MTs have been shown to exert a significant influence on mechanotransduction within the heart and skeletal muscle by enhancing stiffness, which consequently results in aberrant contractile properties (8). The process of detyrosination is facilitated by α-tubulin carboxypeptidases (TCPs) that can be reversed by TTL (18). To investigate the functional implications of the TTL p.G219S, we utilized immunofluorescence imaging and immunoblots against detyrosinated α-tubulin protein in WT and mutant CMs. Our findings reveal a substantial increase in the levels of detyrosinated α-tubulin in mutant CMs compared with WT CMs (Figure 4, A and D). Detyrosinated MTs are linked to desmin at the site of force generation in sarcomeres (19). Recent studies have shown that increased detyrosinated α-tubulin results in increased interaction with desmin (19). Desmin is primarily located at the Z-discs, where it forms a striated pattern and plays a critical role in the mechanical stability of CMs (20). To investigate the effect of elevated detyrosinated α-tubulin on desmin, we performed immunofluorescence imaging and immunoblotting against desmin in patient-specific iPSC-CMs. Our results show that desmin levels were higher in mutant iPSC-CMs than in WT iPSC-CMs (Figure 4, B and C). I In addition, immunoblot analysis was performed to examine the level of extracellular regulated kinase (ERK1/2), a hallmark of pathological hypertrophy (15). ERK1/2 hyperactivation was observed in TTL p.G219S iPSC-CMs (Figure 4D). In addition, we generated a CRISPR/Cas9 gene-edited isogenic line expressing TTL p.G219S that recapitulates the key hypertrophic mechanisms including increased cell surface area, increased levels of detyrosinated α-tubulin, and fetal gene reexpression, similar to that of patient-specific TTL p.G219S iPSC-CMs (Figure 5, A–E). These findings confirm that the TTL mutant CMs exhibit impaired MT stability and altered mechanotransduction.

### Perturbed redox and impaired calcium gene regulation in TTL p.G219S iPSC-CMs.

**Figure 6:**
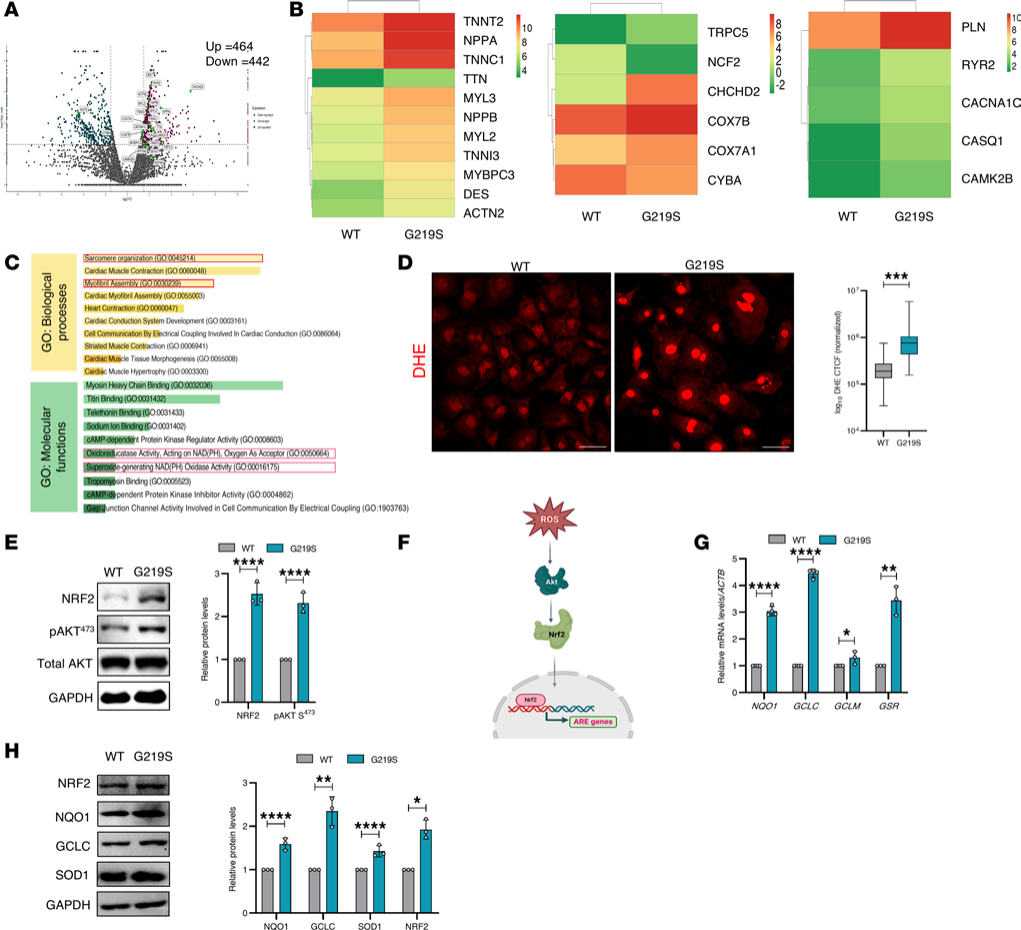
Transcriptome analysis TTL p.G219S iPSCs-CMs showing increased ROS levels and induction of antioxidant response element genes. (**A**) Volcano plot showing the differentially expressed genes in WT versus TTL p.G219S iPSC-CMs (*P* < 0.05); FC denotes fold change. (**B**) A heatmap illustrating the genes that are dysregulated in TTL p.G219S compared with WT iPSC-CMs, with a FC > 1.0. The heatmaps represent hypertrophy-related and structural genes (left), redox homeostasis-related genes (middle), and calcium-related genes (right). (**C**) The dysregulated genes were further analyzed using gene ontology (GO) to identify the biological processes and molecular functions that were significantly affected, with *P* < 0.05. The left section of the GO analysis shows the biological processes, and the right section shows the molecular functions. Red boxes represent relevant processes and functions. (**D**) Representative immunofluorescence of ROS by DHE in WT and TTL p.G219S iPSC-CMs, *n* = 80–90 live cells per group. Red signal indicates ROS. Scale bar: 50 μm. (**E**) Representative immunoblots of WT and TTL p.G219S iPSCs-CMs probed for phospho-AKT (S473) and NRF2 at day 40. (**F**) Schematic depicting the ROS inducted NRF2/AKT pathway leading to activation of ARE genes. (**G**) qPCR analysis of antioxidant response element genes like (*NQO1*, *GCLC*, and *GCLM*). ROS levels were quantified based on intensity, and corrected total cell fluorescence was calculated. (**H**) Representative immunoblots from WT and TTL p.G219S iPSCs-CMs probed for Nrf2 and antioxidant response element proteins NQO1, SOD1, and GCLC quantified on the right. Immunoblot analyses are from same blot or from blots run in parallel with same loading pattern. Values are shown as mean ± SEM with each experiment performed in triplicate. Significance was evaluated by Student’s *t* test or multiple *t* test. *P < 0.05, **P < 0.01, ***P < 0.001, ****P < 0.0001.

To gain a deeper understanding of the molecular mechanisms underlying disease pathology, we performed global RNA-Seq on WT and TTL p.G219S iPSC-CMs. Our RNA-Seq analysis revealed 906 differentially expressed genes (DEGs), including genes involved in sarcomeric organization, mitochondrial functions, redox metabolism, and calcium-handling genes related to contractile function of the heart (Figure 6, A–C, and Supplemental Figure 3A). In addition, we observed that genes associated with cardiac hypertrophy, such as *NPPA*, *NPPB*, *TNNC1*, and *DES*, were markedly upregulated in the mutant compared with WT iPSC-CMs (Figure 6B). We found that genes involved in maintaining redox homeostasis, such as *CYBB* (p91-phox), *NCF2* (p67-phox), *COX7A1*, *COX7B*, *CYBB*, *TRPC5*, and *CHCHD2*, were dysregulated (Figure 6B). Additionally, genes involved in calcium handling, such as *PLN*, *RYR2*, *CAMK2B*, *CASQ*, and *CACNA1C*, were also dysregulated in the TTL mutant iPSC-CMs (Figure 6B). Given that optimal calcium and reactive oxygen species (ROS) levels are essential for maintaining the balance of cardiac signaling networks, the disturbed calcium handling and redox-related genes in mutant iPSC-CMs suggest a pathological condition (Figure 6C).

### TTL p.G219S iPSCs-CMs exhibit oxidative stress.

Recently, MT-induced ROS has been linked to heart failure (21). To evaluate the role of ROS in disease pathogenesis, we performed a live cell staining assay using dihydroethidium (DHE) to assess the levels of ROS in WT and TTL p.G219S iPSC-CMs. The immunofluorescence images show a higher intensity of ROS in the TTL p.G219S variant iPSC-CMs compared with the WT iPSC-CMs at day 30, suggesting a role of oxidative stress (Figure 6D).

We subsequently analyzed the levels of NRF2-AKT (a master regulator of the redox pathway), which activates the antioxidant response element (ARE) genes (22). Our analysis revealed a significant increase in the expression levels of NRF2, AKT, and nuclear translocation of NRF2 in TTL p.G219S CMs compared with WT CMs (Figure 6, E and F, and Supplemental Figure 3B). Moreover, we observed that genes involved in the ARE, such as *GCLC* (glutamate-cysteine ligase catalytic subunit), *NQO1* [NAD(P)H dehydrogenase (quinone 1)], *GCLM* (glutamate-cysteine ligase modified subunit), and *GSR* (glutathione reductase), were upregulated in TTL p.G219S relative to WT iPSCs-CMs, as shown in quantitative PCR (qPCR) and immunoblots (Figure 6, G and H). These findings confirm the presence of oxidative stress in these CMs.

### TTL p.G219S induced detyrosinated α-tubulin, and ROS levels are rescued by parthenolide.

**Figure 7:**
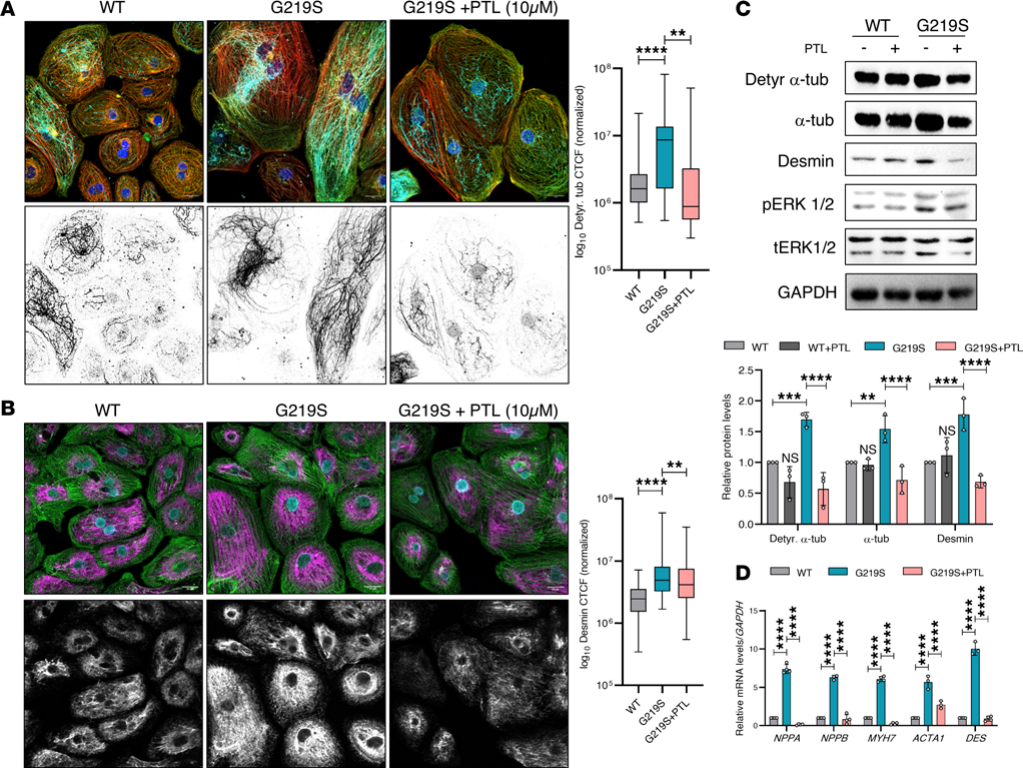
PTL treatment in TTL p.G129S iPSC-CMs normalizes α-tubulin detyrosination. (**A**) Immunofluorescence images of detyrosinated α-tubulin (cyan), α-sarcomeric actinin (green), phalloidin (red), and Hoechst (blue) were captured in WT, untreated, and treated TTL p.G219S iPSCs-CMs with PTL (10 mM for 2 hours). The WT, regardless of treatment, showed no significant differences. WT serves as a comparative control. Lower panel is the grayscale of the detyrosinated α-tubulin of the same image of the upper panel. Scale bar: 20 μm. (**B**) Immunofluorescence images of desmin (magenta), α-sarcomeric actinin (green), and Hoechst (cyan) in WT, untreated, and treated TTL p.G219S iPSCs-CMs with PTL (10 μM). Lower panel is the grayscale of the desmin of the same image of the upper panel. Levels of detyrosinated α-tubulin and desmin were identified based on intensity, and corrected total cell fluorescence was measured (*n* = 50–60 cells per group). Scale bar: 20 μm. (**C**) Immunoblots from the lysates from WT and TTL p.G219S iPSCs-CMs with and without with 10 μM PTL treatment, with quantifications below. (**D**) qPCR analysis of hypertrophic genes (fetal gene markers) in WT, treated, and untreated TTL p.G219S iPSC-CMs with PTL. Values are shown as mean ± SEM with each experiment performed in triplicate. Significance was evaluated by 1-way ANOVA with post hoc Tukey’s test. **P < 0.01, ***P < 0.001, and ****P < 0.0001.

TTL reverses the detyrosination induced by a TCP (6, 18). To ascertain whether the reduction in detyrosinated α-tubulin levels due to decreased TTL activity, we treated WT and TTL p.G219S iPSC-CMs with parthenolide (PTL), an inhibitor of detyrosination (23). Following the acute treatment of PTL (10 mM for 2 hours), we observed a substantial decrease in detyrosinated α-tubulin levels in the TTL p.G219S compared with untreated TTL p.G219S iPSC-CMs (Figure 7, A and C). Subsequently, as expected, there was also a significant decrease in the levels of desmin (Figure 7, B and C). These data suggest that the increased detyrosinated α-tubulin is a result of reduced TTL p.G219S activity. We also observed a significant decrease in the expression of hypertrophy markers such as pERK1/2 (Figure 7C) and fetal gene markers (Figure 7D) upon PTL treatment in TTL p.G219S versus untreated TTL p.G219S iPSCs-CMs.

**Figure 8:**
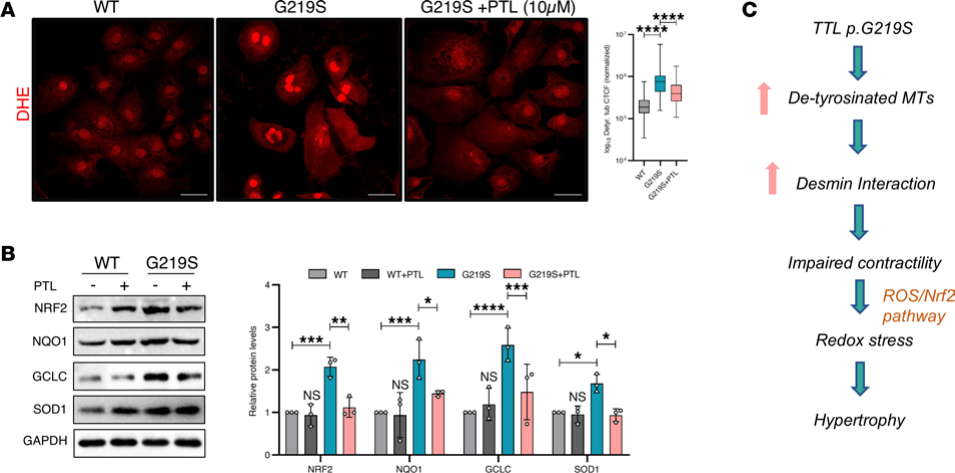
PTL treatment rescued redox stress in TTL p.G219S iPSC-CMs. (**A**) Representative immunofluorescence of ROS by DHE in WT and TTL p.G219S iPSC-CMs with and without PTL treatment (10 μM for 2 hours) (live cell staining) at day 30. Scale bar: 50 μm. The WT, regardless of treatment, showed no significant differences. WT serves merely as a comparative control. ROS levels were quantified based on intensity, and corrected total cell fluorescence was calculated as shown on the right. For DHE staining, *n* = 80–90 cells per group were used for analysis. (**B**) Representative immunoblots results probed for NRF2 and antioxidant response element genes (NQO1, SOD1 and GCLC) upon treatment with PTL (10 μM) in TTL p.G219S iPSC-CMs at day 40. Immunoblot analyses are from the same blot or from blots run in parallel with same loading pattern. (**C**) Proposed model of TTL p.G219S induced hypertrophy. Values are shown as mean ± SEM with each experiment performed in triplicate. Significance was evaluated by 1-way ANOVA with post hoc Tukey’s test. *P < 0.05, **P < 0.01, ***P < 0.001, and ****P < 0. 0001.

Following PTL treatment, we observed a significant reduction in ROS levels and NRF2 and ARE proteins (NQO1, GCLC, and SOD1) in treated compared with untreated TTL p.G219S iPSC-CMs (Figure 8, A and B). Taken together, these data suggest that TTL p.G219S showed reduced activity, resulting in increased detyrosinated α-tubulin, consequently leading to oxidative stress culminating in cardiac hypertrophy (Figure 8C).

## Discussion

TTL catalyzes the addition of a tyrosine residue to the detyrosinated α-tubulin in an ATP-dependent manner (7, 17, 23). This modification is a reversible posttranslational modification of α-tubulin and is essential for maintaining MT dynamics. In this study, we discovered a gene variant in TTL (p.G219S) in a patient with HCM from South Asian India. Notably, more than half of patients with HCM have unknown causes, and the South Asian patients with HCM have been poorly studied. Neither of the proband’s parents carried this mutation, suggesting that the variant arose de novo. The region containing the variation is highly conserved across species, and the variant has been predicted to be pathological using various in silico analyses. Detyrosinated MTs have been reported to interact with desmin at the force generating regions of sarcomeres (19). When detyrosination is decreased, these MTs detach from the sarcomeres and experience less buckling during muscle contraction (19). Thus any alterations in the detyrosination of MTs leads to myocardial dysfunction, which can be accompanied by mechanical and pathological stress such as disrupted redox homeostasis and calcium handling (8). In contrast, chronic activation of α-tubulin tyrosination has been shown to be cardiac protective in mice and human models (24).

The TTL enzyme interacts with the detyrosinated α/β-tubulin dimer to form a complex, and the interaction between the 2 is dependent on a specific conformation of the dimer (7, 16). This conformation is lost when α/β-tubulin dimers are incorporated into MTs, which prevents TTL from tyrosination of MTs (17, 25). Our MD simulations of the α/β-tubulin dimer and mutant TTL protein revealed alterations in the conformation of the mutant protein near its catalytic region. This alteration may reduce its ability to attach to the α/β-tubulin heterodimer compared with that of the WT protein. Our in vitro tyrosination assay confirmed this in silico finding, showing reduced activity of the TTL p.G219S mutant, which resulted in increased detyrosinated α-tubulin.

By generating iPSCs from the TTL p.G219S patient’s peripheral blood mononuclear cells (PBMCs) and differentiating them into CMs, we showed an increase in cell surface area, as expected in HCM, along with the upregulation of fetal gene reexpression and activated RAS-MAPK signaling, as indicated by higher levels of phospho-ERK1/2 in iPSC-CMs (15). Strikingly, we observed 2 distinct cell states across time: one with increased sarcomeric organization, which represents a hypertrophic response (day 30), and the other with decreased sarcomeric organization (day 40), which might represent different stages of heart failure phenotypes (26). These findings suggest that TTL p.G219S patients are at a higher risk of transition from HCM to heart failure.

We also observed an increase in the detyrosinated α-tubulin form in patient-specific CMs, confirming the reduced activity of the TTL p.G219S. Detyrosinated MTs in CMs offer mechanical resistance, hindering the contraction of CMs and resulting in reduced cell biomechanics, contractility, and mechanotransduction (8). A prominent characteristic of increased stability of MTs, which happens when detyrosinated tubulin is increased in CMs, is an accumulation of cytoskeletal proteins, especially intermediate filaments like desmin (8). These alterations may initially serve an adaptive purpose, perhaps safeguarding a heart under severe mechanical strain (27). However, as they advance, these changes might become maladaptive by further increasing the MT stability, thereby impairing sarcomeric contraction and relaxation (27). In this study, we found that desmin levels are significantly higher in mutant iPSC-CMs than in WT iPSC-CMs, which is reversed upon treatment with PTL, suggesting that the increased levels of desmin are due to the increased detyrosination of MTs in the mutant.

A growing body of evidence suggests that oxidative stress is implicated in cardiac dysfunctions associated with MTs (8). Specifically, the role of ROS regulatory components, such as NADPH oxidases (e.g., NOX2 and NOX4) and their downstream pathways (NRF2/AKT axis) in the progression of cardiac hypertrophy is well documented (22). In our study, we observed a significant increase in ROS regulatory components (NRF2/AKT) in mutant CMs compared with WT cells at day 30, confirming increased oxidative stress. Subsequently, at day 40, ROS levels were not detectable, suggesting a shift toward reductive stress, which is shown to be a risk factor involved in transition of hypertrophy to heart failure (28).

The interplay between increased ROS and calcium signaling in the heart is a complex yet crucial aspect of cardiac physiopathology (29). ROS-led oxidation adjusts the sensitivity of calcium handling proteins leading to an elevated rate of calcium (Ca^2+^) sparks and an improvement in Ca^2+^ signaling (29, 30). ROS can directly influence the activity of Ca^2+^-handling proteins such as SERCA2a, thereby affecting Ca^2+^ release and reuptake kinetics within the CM (31). Additionally, ROS can induce posttranslational modifications of key Ca^2+^-handling proteins (32), altering their function and contributing to aberrant Ca^2+^ handling observed in conditions such as heart failure and ischemia-reperfusion injury. In line with this, we observed dysregulation of Ca^2+^-handling proteins, including SERCA2a and PLN. Mutant CMs showed lower SERCA2a expression, indicating reduced Ca²^+^ reuptake into the sarcoplasmic reticulum and affecting contractility. The activity of SERCA2a is regulated by PLN. The contraction cycle is contingent upon the ratio of SERCA2a to PLN (33, 34). Thus, an imbalance in the SERCA2a/PLN ratio, as observed in TTL p.G219S iPSC-CMs, contributes to contractile dysfunction.

In our study, we used PTL to investigate the influence of TTL p.G219S induced detyrosination–related pathogenesis. After administering PTL, we observed a reduction in the hypertrophy response by decreasing the levels of detyrosinated α-tubulin in TTL p.G219S compared with untreated TTL p.G219S iPSC-CMs. This may be attributed to the reduced activity of the TTL variant. Furthermore, PTL treatment reduced the levels of ROS and oxidative stress–induced antioxidant response pathways. These results imply that disruption of the redox balance is the primary pathway that leads to the hypertrophic response caused by the TTL p.G219S variant.

Our study presents a comprehensive framework that sheds light on the mechanisms underlying the hypertrophy of TTL p.G219S iPSC-CMs. We found that a decrease in TTL activity results in the accumulation of detyrosinated α-tubulin in CMs, which causes a redox imbalance and impairs contractile function. Combined with the genetic and biochemical data, our findings offer strong evidence that the *TTL* variant plays a crucial role in the development of HCM. Although TTL is known to regulate MT dynamics, our study represents the first evidence to our knowledge that its alteration can lead to HCM in humans.

## Methods

### Sex as a biological variable.

Our study used an available male patient-specific iPSC line, where the TTL variant was identified.

### Whole exome sequencing.

We sequenced and analyzed the P1 exome using our previously published exome analysis pipeline (9). In brief, paired-end 100 bp reads (100 × coverage) were used, and 6 GB data were obtained. The average coverage of the target region of the capture kit was 90%, over 30×. Low-quality reads were filtered, and adapters were removed using Trimmomatic. The exome was first mapped onto the human reference genome (GRCh38) using the Burrows-Wheeler aligner V.0.7. Variant calling was performed using HaplotypeCaller from Genome Analysis Tool Kit V.3.4. Variants were annotated using the web interface of the ANNOVAR software. Pathogenic or deleterious variants were classified using in silico tools, including Polymorphism Phenotyping v2 (PolyPhen2) (35), Sorting Intolerant From Tolerant (SIFT) (36), Combined Annotation Dependent Depletion (CADD) (37), Mutation Taster (38), Mendelian Clinically Applicable Pathogenicity (M-CAP) (39) Score, Protein Variation Effect Analyzer (PROVEAN) (40), MetaLR, Domain Adversarial Neural Network (DANN) (41), and functional analysis through hidden markov model-based on multiple kernel learning (Fathmm-MKL) (42). The majority of in silico tools (6 of 9) should predict a variant to be deemed as pathogenic or deleterious and were reviewed against the criteria defined by the American College of Medical Genetics.

The following variables were considered as critical for ranking the pathogenic variants: (a) coding regions; (b) rare variants ≤ 0.1% (0.001) MAF; (c) ultrarare variants ≤ 0.01% (0.0001) MAF; and (d) potentially novel variants in the public human population genome reference data sets with various ethnicities. The following reference datasets were used to estimate allele frequency: (a) Mixed control datasets (gnomAD); (b) disease data sets (Genotype to Mendelian Phenotype [Geno2MP v2.4]); (c) Indian and White Wellderly control data sets including healthy aging Indian exomes (www.instem.res.in/IndiCardiome) and the Wellderly data set (43); (d) South Asia–specific Indian control data sets including Genome Asia 100 K (44), IndiGenomes, and South Asian (Indian) healthy controls (4). The cardiac-specific expressing genes were obtained from the human protein atlas (45) and single cell genomics data (46) and include all the known cardiac development, physiology, and cardiomyopathy-associated genes. The presence of pathogenic variants in all cardiac-related genes were filtered and analyzed.

### Generation of iPSCs.

Patient-specific PBMCs were isolated using the density gradient method with Histopaque (MilliporeSigma, 1077). iPSCs were generated using the Cytotune2.0 Sendai virus kit (Invitrogen, A16517).

Initially, cells were seeded in a well of 24-well plate at a cell count of 3.5 × 10^5^ per μL with 95% viability on day –4, in complete PBMC media. PBMC medium consisted of complete StemPro-34 medium (Invitrogen, 10639011) supplemented with the appropriate cytokines (SCF c-kit 100 ng/mL [Invitrogen, PHC2115], FLT-3 100 ng/mL [Invitrogen, PHC9414], IL-3 20 ng/mL [Invitrogen, PHC0034], and IL-6 20 ng/mL [Invitrogen, PHC0064] at their final concentration). On day 0, cells were transduced with Sendai reprogramming vectors at appropriate MOIs. The volume of virus used was calculated using the following equation: V= (MOI [CIU/cell] × number of cells) / titer of virus (CIU/cell) × 10^–3^ (mL/μL), where V = volume of virus used for reprogramming.

On day 1, the medium was replaced with PBMC medium to remove the viral titer. On day 3, cells were plated onto vitronectin-coated plates in complete StemPro-34 medium without cytokines. On day 7, transitioning began in Essential-8 medium. From day 8 to day 28, the medium was changed every day and was checked for iPSCs colony emergence. After stable proliferation of iPSCs colonies, cells were passaged and expanded for characterization and differentiation with the equations in Supplemental Methods.

### TTL p.G219S isogenic line generation using CRISPR/Cas9 gene editing.

An isogeneic line was generated using CRISPR-Cas9 system by replacing the WT with the mutant nucleotide (c.655G>A) in a healthy control iPSC line. RNP complex was formed with 1 mg Cas9 protein (Invitrogen, A36498), 5 mM sgRNA (Synthego, Target site: TAATATCTACCTCTATAGAG) and 5 mM single-stranded oligodeoxynucleotide homology directed repair template (IDT, Sequence-gcagAAGCTGGGTCTTGGTGGATCATCAGTATAATATCTACCTCTATAGAGAGaGTGTGCTTCGGACTGCTTCAG AACCATATCATGTTGATAATTTCCAAGACAAAACCTGCC) and electroporated in 100,000 cells using Neon transfection kit 10 mL (Invitrogen, MPK1025R) with the following pulse settings: 1150 V, 30 ms pulse width, 1 pulse. Two days after electroporation of cells, single-cell seeding was done in 96 wells, and cells were collected once appropriate colony size was reached. DNA was isolated using DNAzol (Invitrogen, 10503027), and target region was amplified using PCR. Purified PCR products were sent for sanger sequencing for mutation confirmation.

### Embryoid body formation.

Embryoid bodies are aggregates of ESCs or iPSCs that can form a 3D structure in a differentiated manner. Over the course of 2–4 days, all 3 germ layers ectoderm (outer layer), mesoderm (middle layer), and endoderm (inner layer) are present in differentiated embryoid bodies. For embryoid formation, after passaging iPSCs with ReleSR (Stem Cell Technologies, 100-0484) for 3 minutes, cells were removed by gentle pipetting into a 15 mL Falcon tube containing 5 mL of iPSC culture media, Stemflex (Thermo Fisher Scientific, A3349401), and pelleted at 200 rcf for 5 minutes. The supernatant was removed, the cell pellet was resuspended in 1 mL of Stemflex medium, and 10 μL of cells were taken out for cell counting. Cells were mixed with 10 μL of trypan blue and counted using a Countess 3 automated cell counter. Approximately 30,000 cells were seeded into each well of an ultra-low–attachment 24-well plate with 500 μL of Stemflex media. Cells were then allowed to form aggregates for next 24–48 hours and then taken out for RNA isolation.

### Germ layer marker analysis.

Isolated RNA from fully formed embryoid bodies was converted to cDNA using Verso cDNA synthesis kit (Thermo Fisher Scientific, AB1453A) and the generated cDNA was subjected to PCR as a template against different germ layer markers like *GATA4* (mesoderm), *NODAL* (endoderm), *SOX1* and *NES* (ectoderm), and *RNU6-1* (housekeeping gene).

### qPCR.

Total RNA was extracted from iPSC-CMs. The cDNA was synthesized using 1 mg RNA and Verso cDNA synthesis kit (Thermo Fisher Scientific, AB1453A). cDNA was amplified using real-time PCR. Data were analyzed using the 2^ΔΔCT^ method. Relative fold changes in gene expression were normalized to *RNU6-1* or *GAPDH*. All the relevant primers used in the study are outlined in Supplemental Table 1.

### Karyotyping.

iPSCs generated at 50% confluence were karyotyped using the Giemsa (G-banding) cytogenetic staining technique to detect condensed chromosomes.

### Characterization of pluripotency.

Pluripotency status of the generated iPSCs were checked using immunocytochemical staining against different pluripotency markers like Oct3/4 (Santa Cruz Biotechnology Inc., sc-5279), SSEA3 (Santa Cruz Biotechnology Inc., sc-21703), SSEA4 (Santa Cruz Biotechnology Inc., sc-21704), Tra 1-60 (Santa Cruz Biotechnology Inc., sc-21705), and Tra 1-81 (Santa Cruz Biotechnology Inc., sc-21706). The protocol mentioned above for immunocytochemical staining was also followed in this experiment.

### CM differentiation.

Initially, patient-specific iPSCs and control line (WT) were acclimatized to mTeSr medium (STEMCELL Technologies, 100-0276), and cells were seeded in Matrigel-coated (Merck Corning, CLS354234) 12-well plates for differentiation. After reaching 70%–80% confluency, differentiation was started with induction by 4 μM CHIR99021 (Sigma-Aldrich, SML1046) for 48 hours followed by 5 μM IWP2 (Wnt inhibitor) (Sigma-Aldrich, I0536) for 2 days in cardiac differentiation medium (RPMI1640 with Glutamax [Thermo Fisher Scientific, 61870036], 213 μg/mL ascorbic acid [Sigma-Aldrich, A92902], 500 μg/mL human serum albumin [Sigma-Aldrich, A1887], and B27 minus insulin [Thermo Fisher Scientific, A1895601]). Cells were maintained until day 7 in cardiac differentiation media and were then replaced with cardiac maintenance media (RPMI1640 with Glutamax and B27 supplement [Thermo Fisher Scientific, 17504044]) for the next 10 days. On day 15 and 16, cells were transitioned to lactate medium (Sodium L-lactate [Sigma-Aldrich, 71718], RPMI1640 minus Glucose [Thermo Fisher Scientific, 11879020], 213 μg/mL ascorbic acid, and 500 μg/mL human serum albumin) for 4 days for purification of CMs. After recovery of cells in cardiac maintenance media for 4 days, cells were passaged and plated onto freshly coated Matrigel plates/dishes and with cardiac maintenance media (RPMI1640 with Glutamax, B27 supplement, and 5% knockout serum [KOSR]) until day 30 or 40.

### Immunocytochemical staining of iPSC-CMs.

iPSC-CMs from the patient and the control line were dissociated and seeded on Matrigel-coated Ibidi confocal dishes (Ibidi, 81156). Cells on the dishes were washed 3 times with PBS and then fixed with 4% paraformaldehyde (Sigma-Aldrich, 158127) for 15 minutes at room temperature (RT). Again, coverslips were washed thoroughly with PBS 3 times and permeabilized afterward with 0.3% Triton-X100 (Sigma-Aldrich, 93443) for 15 minutes at RT. Cells were then blocked with 3% BSA, 1% NGS, 0.3% Triton-X100 for 1 hour at RT. Primary antibody, ACTN2 (Invitrogen, MA1-22863, dilution 1:500), detyrosinated α-tubulin (Abcam, ab48389, dilution 1:500), desmin (Thermo Fisher Scientific, MA5-32068, dilution 1:500), NRF2 (Cell Signaling Technology, 12721, dilution 1:100), and cardiac troponin T (Thermo Fisher Scientific, MA5-12960, dilution 1:100) were added onto dishes overnight at 4°C with respect to experiments requirements. The next day, dishes were washed 3 times with PBST (PBS+0.1% Tween 20), and then secondary antibody was added to the dishes (Alexa Fluor 488, 647 in accordance with the required experiment) for 1 hour at RT and then washed 3 times with PBST. After this, Phalloidin 546 (Thermo Fisher Scientific, A22283, dilution 1:400) along with Hoechst (Thermo Fisher Scientific, 62249, dilution 1:10,000) was added to the dishes for 1 hour at RT and then washed with PBST 3 times and imaged on an Olympus FV3000, 20×/60× NA 1.40 oil immersion objective.

### Muscle motion analysis.

The Muscle Motion Analysis procedure was performed using the plug-in Muscle Motion Analysis available in ImageJ (NIH). The first step is to import video files that include muscle movement data into ImageJ. To reduce the effects of motion artifacts, preprocessing was conducted, such as brightness/contrast adjustment and stabilization of videos using a stabilizer. Subsequently, the Muscle Motion Analysis plug-in was installed and activated through the plug-ins menu. Within these iPSC-CMs are defined ROIs, while the frame rate and analysis method were set according to how the experimental setup was done. This thereafter ran the plug-in for tracking ROIs that were chosen throughout all video sequences. The results were generated and evaluated, which included motion trajectories as well as displacement maps. The analysis results also provided quantitative data, such as displacement values and velocity profiles, that could be extracted from the experiment’s outcome. Consequently, these findings were interpreted within the scope of the research objectives to understand their significance in terms of physiological function or experimental outcomes. To ensure the reliability and accuracy of both the methodology and results, validation procedures were carried out.

### Cell lysate preparation.

Cells were lysed in RIPA buffer (50 mM Tris [pH 8.0], 150 mM NaCl, 10 mM EDTA, 10% glycerol, 1% Triton X-100, 0.1% SDS, 1× protease inhibitor cocktail). Protein concentrations of cell lysates were measured using the BCA method, and approximately 15–20 μg of total protein was loaded onto gels and separated by SDS-PAGE.

### Immunoblotting.

Protein gels (SDS-PAGE) were transferred onto polyvinylidene difluoride (PVDF) membranes. Membranes were blocked in 3% BSA and incubated with primary antibodies overnight at 4°C. Membranes were then incubated with appropriate secondary antibodies conjugated to horseradish peroxidase (Invitrogen, 31460 and 31431), and signal intensities were visualized by chemiluminescence.

The antibodies used are as follows: Nrf2 (Cell Signaling Technology, 12721), SOD1 (Thermo Fisher Scientific, MA1105), NQO1 (Thermo Fisher Scientific, MA1-16672), GCLC (Thermo Fisher Scientific, MA5-26346), TTL (Thermo Fisher Scientific, PA5-66871), tyrosinated α-tubulin (Sigma-Aldrich, SAB4200776), detyrosinated α-tubulin (Abcam, ab48389), α-tubulin (Sigma-Aldrich, T9026), phospho-ERK1/2 (Cell Signaling Technology, 9101), ERK1/2 (Cell Signaling Technology, 9102), GAPDH (Thermo Fisher Scientific, MA5-15738), phospho-AKT (Cell Signaling Technology, 4060), AKT (Cell Signaling Technology, 4691), Desmin (Sigma-Aldrich, D1033), SERCA2 (Cell Signaling Technology, 9580), phospho-PLN (Cell Signaling Technology, 8496), and PLN (Cell Signaling Technology, 14562).

### Purification of recombinant TTL proteins.

Human TTL (hTTL) variants (WT and G219S) with a C-terminal 6XHis-tag were overexpressed in *E*. *coli* (bl21) cells and purified as described previously with some modifications. Transformed *E*. *coli* cells were induced with 0.5 mM IPTG overnight at 27°C to initiate protein expression. After induction, cells were harvested by centrifugation at 100,000*g* for 10 minutes at 4°C, and the resulting cell pellet was washed 2 times with PBS. The pellet was resuspended in cold lysis buffer and sonicated on ice for 10 cycles. The soluble cell lysate was purified using a His Trap column. The soluble fraction was equilibrated to a final concentration of 10 mM imidazole before loading onto the column. The column was thoroughly washed using wash buffer, and the bound hTTL was eluted with elution buffer containing 500 mM Imidazole. Following elution, enriched hTTL fractions were pooled and further purified using a Sephadex200 16/60 column connected to an FPLC purification system. The purity and concentration of the protein were determined using SDS-PAGE.

### Tubulin tyrosination assay.

Tubulin was purified from the goat brain using 2 cycles of polymerization and depolymerization. CPA treatment of purified goat brain tubulin was performed. A reaction mixture containing purified goat brain tubulin with 20% glycerol, 2 mM GTP, and 2.5 μg/mL of pancreatic CPA was prepared in 1× BRB80 (see Supplemental Methods). Briefly, the mixture was incubated on ice for 10 minutes, and the reaction was initiated by incubation at 37°C for 20 minutes. After incubation, the reaction was immediately stopped by adding 20 mM DTT, and the MTs were further allowed to polymerize for 10 minutes at 37°C. Polymerized MTs were then pelleted at 100,000*g* for 40 minutes at 37°C and were further resuspended in ice-cold BRB80.

A reaction mixture (10 μL) containing CPA-treated tubulin (2.5 μM) in 50 mM MES/K, 100 mM KCl, 10 mM MgCl_2_, 5 mM DTT, 2.5 mM ATP, and 0.2 mm l-tyrosine was used to analyze the tyrosination abilities of TTL variants. The mixture was incubated with TTL at a molar ratio of 1:0.0002 for different time points (0, 5, 10, 15, 20, and 30 minutes) at 37°C in a circulating water bath. The enzymatic reaction was stopped using 2× SDS-PAGE Sample Buffer. The resulting samples were subsequently analyzed using Western blotting and were probed using anti–Tyr-α-tubulin (1:5,000) or anti–α-tubulin (1:5,000), followed by anti–mouse or –rabbit peroxidase–conjugated secondary antibody (1:5,000) (see Supplemental Methods). ImageJ was used to quantify the relative intensities of the Western blot bands.

### MD simulation.

MD simulations of α-tubulin-TTL complexes (both WT [ref. 16] [PDB ID: 4IHJ] and G219S mutant) were performed using the Charmm36 force field within the GROMACS 2019 framework. All the simulations involved positioning of the α-tubulin-TTL complexes at the center of an octahedral box with a distance of 15 Å from the boundary walls surrounded by transferable intermolecular potential with 3 points model water molecules for solvation under periodic boundary conditions. The neutralization of the complexes was carried out by adding an appropriate amount of sodium and chlorine ions, and a topology file was generated. The model systems were subjected to energy minimization and subsequent equilibration using a canonical ensemble (amount of substance [N], volume [V], and temperature [T]) followed by isothermal-isobaric ensemble (Normalization Process Theory). Finally, the simulation was performed for 100 ns.

### Global RNA-Seq and analysis.

RNA was isolated from the respective CMs using the Trizol RNA extraction method. Sequencing was performed at the institutional sequencing facility. RNA integrity was checked using a Bioanalyzer, and samples with RNA integrity number above 9 were used for analysis. cDNA was prepared according to the manufacturer’s instructions using the NEBNext Ultra Directional RNA Library Prep Kit for Illumina (catalog NEB-E7760). For mRNA enrichment, the polyA selection method was employed. Next-generation sequencing of libraries was performed on the Illumina HiSeq 2500 platform for 1 × 100 bp at ~30 million to 35 million reads per sample.

Sequencing data were trimmed for adaptor sequences and low-quality reads (~15 bp) using Trim Galore! and mapped to GRCh38 using TopHat. Paired-end reads obtained from both ends of the same RNA fragment were examined, and paired reads ≥ 35 bp were retained. DEGs between the samples were identified using Cufflinks/Cuffdiff software with parameters: Log_2_ fold change ± 1.0 and adjusted *P* < 0.05. Gene ontology of biological processes (using Enrichr) was used to show the network of significantly enriched biological processes and pathways.

### Cell size analysis.

To elucidate the cell size, ImageJ software was employed. To retrieve pertinent cellular information from the confocal pictures, the captured multistack images were stacked into 1 image, and the projection with the highest intensity was recovered, which resulted in an improvement in the visibility of cellular structures; then, all the channels were split away. Noise was removed up to a 2-pixel size and 50 μm range. After that, particle analysis was carried out to accurately quantify the cell surface area. Effective tracking of regions of interest was made possible by the administration of segmented cells through the ROI Manager.

### ROS measurement and analysis.

Before staining, the CMs were seeded on suitable culture dishes and allowed to adhere and grow into the ideal morphology. To measure ROS, DHE (Sigma-Aldrich, 309800) was employed. DHE powder was dissolved in dimethyl sulfoxide (DMSO) to form a stock solution at a concentration of 5 mM. To obtain a working concentration of 5 μM, the stock solution was diluted in PBS. The cells were initially rinsed with PBS and then exposed to DHE staining solution at 37°C in the dark for 30 minutes. This incubation period allowed DHE to penetrate cells and undergo oxidation by ROS.

After the incubation period for staining, the staining solution was carefully pipetted out, and the cells were gently rinsed with PBS to eliminate any surplus dye. The cells were subsequently treated with 4% paraformaldehyde solution for 5 minutes at RT to maintain the integrity of the staining pattern. The stained cells were imaged using a confocal microscope that had the necessary filter sets for DHE, with excitation/emission wavelengths of 488/610 nm. Optimal exposure settings were employed to capture a balanced range of fluorescence intensities, avoiding overexposure. These parameters were consistently maintained for all samples.

### Intensity measurement of confocal images.

To measure the intensity of certain biomolecules in the cell upon staining and confocal imaging, a selection was first made using ImageJ. Then, by using the “Analyze” dropdown option, the “set measurements” option was picked, after which “area integrated density” and “mean grey value” were selected. After this, the “measure” option was selected, which gave the intensity of the area selected. Similarly, a region near the cell with no fluorescence was selected, and the intensity was measured. This provided “background.” With these 2 measurements, the corrected total cell fluorescence (CTCF) was calculated using the following formula: CTCF = Integrated density – (Area of selected cell × mean fluorescence of the background).

### Statistics.

Depending on the experiments, statistical significance was determined by 2-tailed Student’s t test, 2- tailed multiple t test, and 1-way ANOVA with post hoc Tukey’s test. *P* < 0.05 was considered to be statistically significant.

### Study approval.

This study was approved by the Institutional Ethical Committee of the Institute for Stem Cell Science and Regenerative Medicine (reference no. inStem/IEC-10/001). Informed written consent for clinical data, blood, and/or tissue samples for the study was obtained from the participants. The IRBs of the study centers approved the study protocols.

### Data availability.

All data relevant to the study are included in the article or provided in the Supporting Data Values file. Relevant sequencing data are available at NCBI GEO BioProject id PRJNA1268140. Data are available upon reasonable request from the corresponding author.

## Author contributions

PSD conceived, designed, analyzed, drafted, and reviewed the manuscript. PKJ performed, designed, and analyzed the major functional studies and drafted the manuscript. MS, VH, CJ, and SM were involved in MD simulations, protein purification, and enzyme activity assays. HC performed gene editing and related experiments.

## Supplementary Material

Supplemental data

Unedited blot and gel images

Supporting data values

## Version 1 08/08/2025

Electronic publication
