# Supplementary material for: Tubulin tyrosine ligase variant perturbs microtubule tyrosination, causing hypertrophy in patient-specific and CRISPR gene-edited iPSC-cardiomyocytes
Source: JCI Insight. 2025 Aug 8;10(15):e187942. doi: 10.1172/jci.insight.187942 (PMC12333943; doi:10.1172/jci.insight.187942)
Supplement: Supplemental data [file jciinsight-10-187942-s164.pdf]

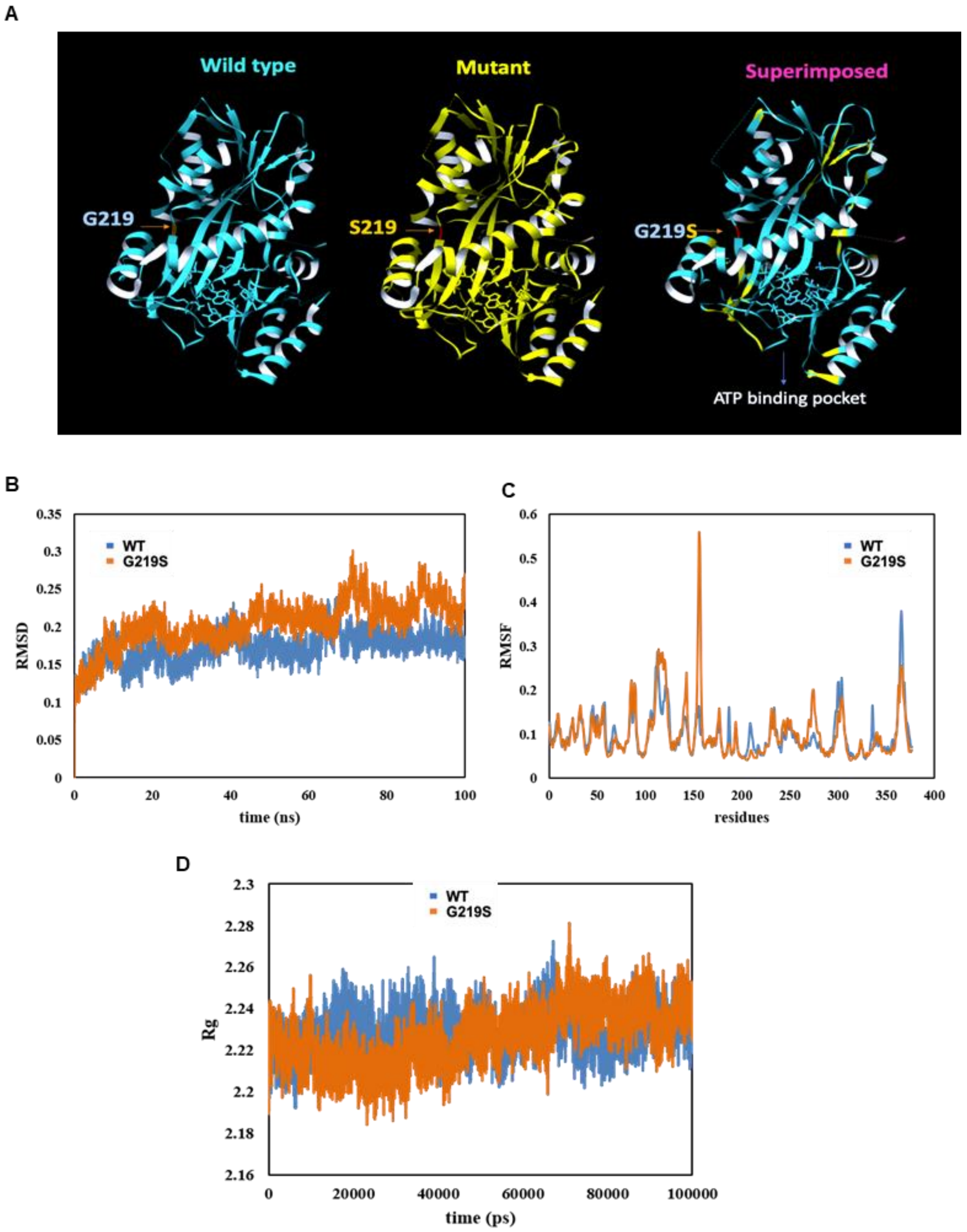

Supplemental Figure 1: Molecular dynamics (MD) simulation of WT and TTL p.G219S.

(A) MD simulation of WT and TTL p.G219S (PDBID: 4IHJ) (bound to the C-terminal tail of the/-tubulin heterodimer and ATP) showing multiple regions that did not overlap upon complex formation. **(B)** Root mean square deviation (RMSD) of WT versus TTL p.G219S. **(C)** Root mean square fluctuations (RMSF) of WT versus TTL p.G219S. **(D)** Radius of gyration (Rg) of WT and TTL p.G219S.

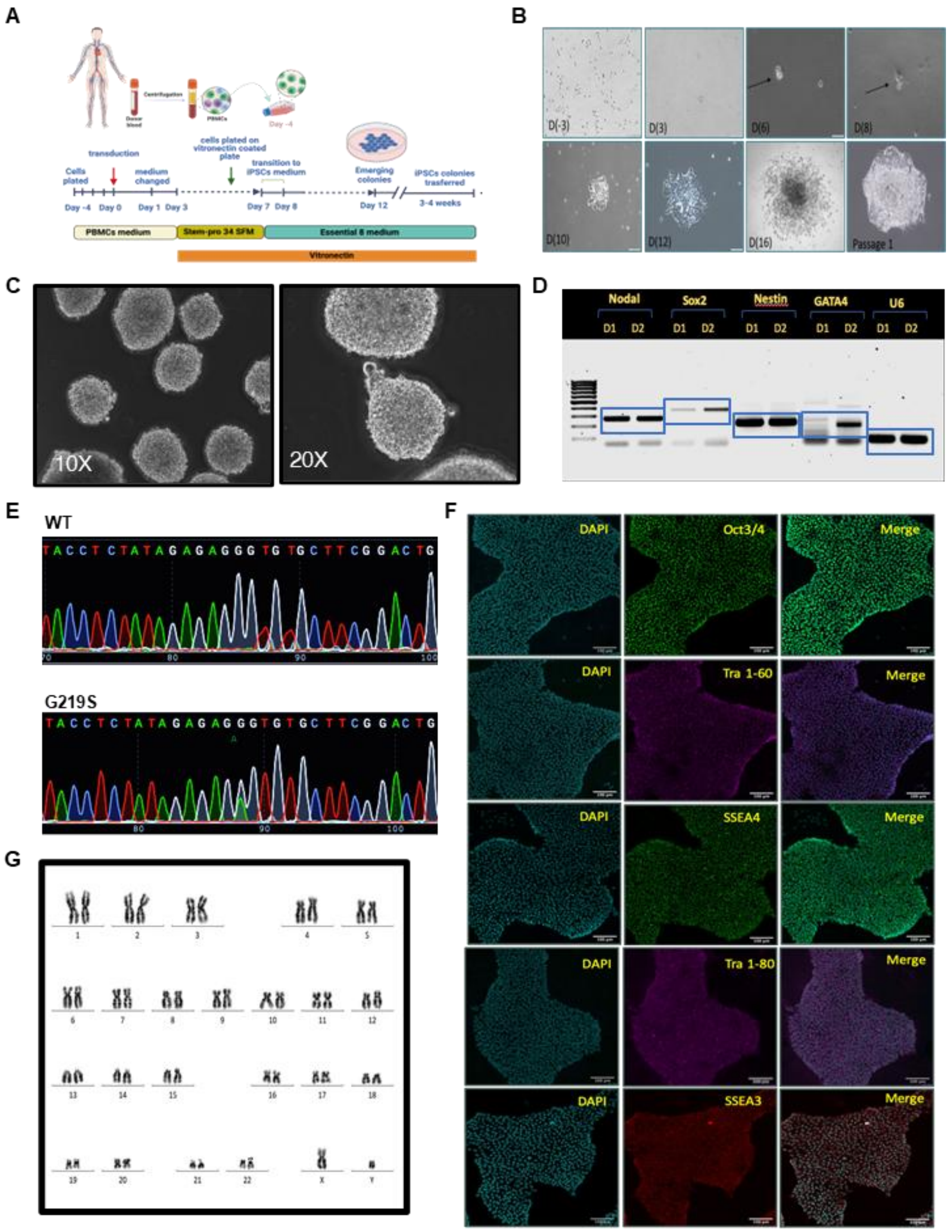

Supplemental Figure 2: Patient-specific iPSCs generation and characterization.

(A) Schematic diagram displaying iPSC generation from TTL p.G219S patient-specific peripheral blood mononuclear cells (PBMCs). (Scale bar: 2000µm). **(B)** Representative images of the different stages of iPSC generation. **(C)** Representative images of embryoid body formation. **(D)** Germ layer markers from total RNA isolated from days 1 and 2 of embryoid bodies. **(E)** Sanger sequencing results from WT and TTL p.G219S iPSCs amplicons. **(F)** iPSC showing normal stem cell morphology and stemness markers, including Oct3/4, Tra 1-60, SSEA4, Tra 1-80 and SSEA3. (Scale bar: 100µm) **(G)** Karyotype analysis of TTL p.G219S iPSCs.

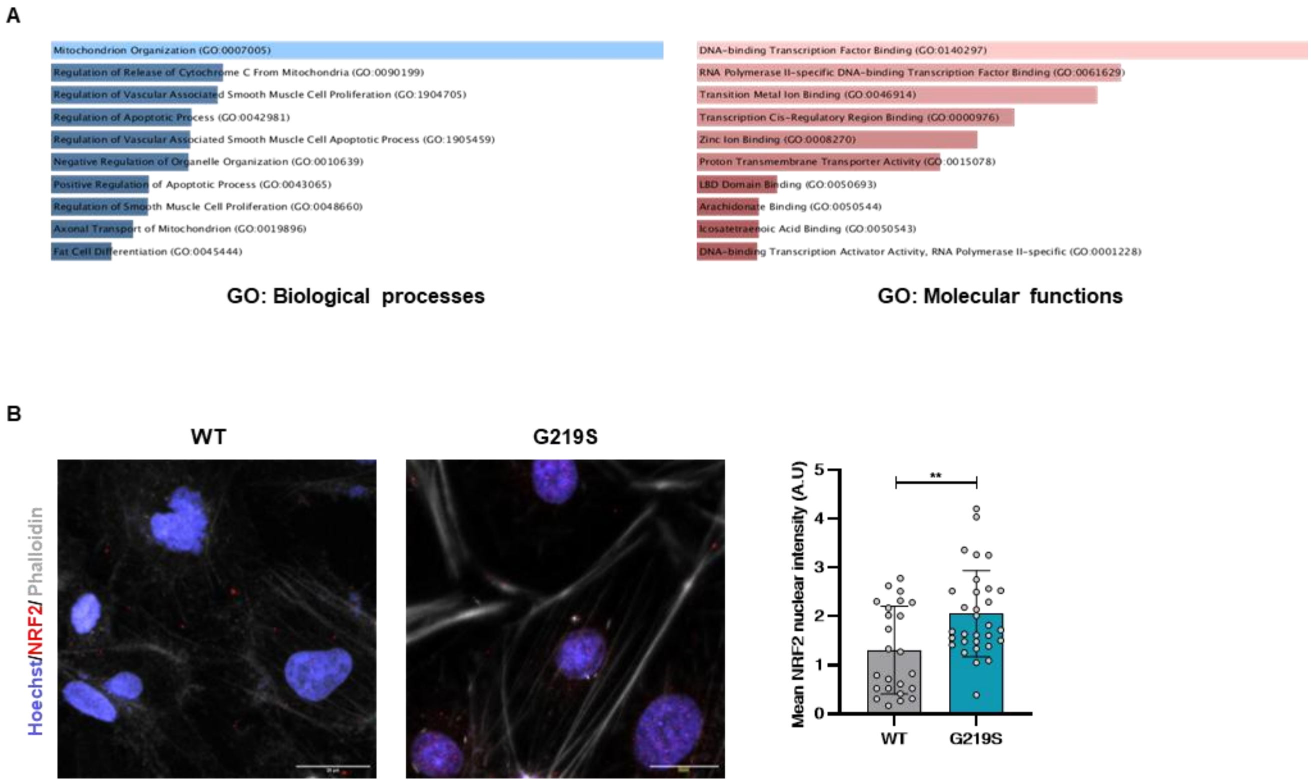

Supplemental Figure 3: TTL p.G219S variant displays dysregulated mitochondrial pathways and NRF2 nuclear localization.

(A) Genes related to mitochondrial function are perturbed as observed in gene ontology (GO), with a p-value < 0.05 compared to WT iPSC-CMs. The left section of the GO analysis shows the biological processes, and the right section shows the molecular functions. **(B)** Representative images of increased nuclear localization of NRF2 in the TTL p.G219S variant compared to the WT iPSC-CMs. Values are shown as mean ±SEM with the experiment performed in triplicate. Significance was evaluated by Student's t-test (**P < 0.01).

Supplemental Table 1: List of primers used in the study

Gene	Forward primer	Reverse primer	Purpose
TTL	CCCACTCCTTACCTACCCCA	GCATAGACGCCAAAGCTCCT	Genotyping (Sanger sequencing)
RNU6-1	ATTGGAACGATACAGAGAAGATTAG	AATATGGAACGCTTCACGAAT	House-keeping control
GATA4	TCCAAACCAGAAAACGGAAG	CTGTGCCCGTAGTGAGATGA	iPSC characterization
NES	AACAGCGACGGAGGTCTCTA	TTCTCTTGTCCCGCAGACTT	iPSC characterization
NODAL	AGACATCATCCGCAGCCTACA	GACCTGGGACAAAGTGACAGTGAA	iPSC characterization
SOX1	GGGAAAACGGGCAAAATAAT	CCATCTGGGCTTCAAGTGTT	iPSC characterization
ACTA1	TCTCACCGACTACCTGATGAA	AGCACAGCTTCTCCTTGATG	Hypertrophy marker
MYH7	TGAAGGAGGACCAGGTGAT	GTAGCGATCCTTGAGGTTGTAG	Hypertrophy marker
NPPA	TTGCTGGACCATTTGGAAGA	GCTTCTTCATTCGGCTCACT	Hypertrophy marker
NPPB	TCCTGCTCTTCTTGCATCTG	GTAACCCGGACGTTTCCAA	Hypertrophy marker
ATP2A2	GAAATGTGTAACGCCCTCAAC	CGACATAGAGGATCAGGAAGTG	Redox stress marker
DES	GGTACAAGTCGAAGGTGTCAG	GGTGTCGGTATTCCATCATCTC	Redox stress marker
GCLC	CCCAAACCATCCTACCCTTT	CATGTTGGCCTCAACTGTATTG	Redox stress marker
GCLM	TCTTGCCTCCTGCTGTGTGATG	TTGGAAACTTGCTTCAGAAAGCAG	Redox stress marker
GSR	GGGACTTGGGTGTGATGAAA	CTTCTGAAGAGGTAGGGTGAATG	Redox stress marker
NQO1	GGGATGAGACACCACTGTATTT	TCTCCTCATCCTGTACCTCTTT	Redox stress marker
PLN	CTCACTCGCTCAGCTATAAGAAG	AGAGAAGCATCACGATGATACAG	Redox stress marker

Supplemental Methods

Methods

Sex as a biological variable. Our study used an available male patient-specific iPSC line, where the TTL variant was identified.

Whole exome sequencing. We sequenced and analyzed the P1 exome using our previously published exome analysis pipeline (9). In brief, paired-end 100 bp reads (100 × coverage) were used, and 6 GB data were obtained. The average coverage of the target region of the capture kit was 90%, over 30×. Low-quality reads were filtered, and adapters were removed using Trimmomatic. The exome was first mapped onto the human reference genome (GRCh38) using the Burrows-Wheeler aligner V.0.7. Variant calling was performed using HaplotypeCaller from Genome Analysis Tool Kit V.3.4. Variants were annotated using the web interface of the ANNOVAR software. Pathogenic or deleterious variants were classified using in silico tools, including Polymorphism Phenotyping v2 (PolyPhen2) (35), Sorting Intolerant From Tolerant (SIFT) (36), Combined Annotation Dependent Depletion (CADD) (37), Mutation Taster (38), Mendelian Clinically Applicable Pathogenicity (M-CAP) (39) Score, Protein Variation Effect Analyzer (PROVEAN) (40), MetaLR, Domain Adversarial Neural Network (DANN) (41), and functional analysis through hidden markov model-based on multiple kernel learning (Fathmm-MKL) (42). The majority of in silico tools (6 of 9) should predict a variant to be deemed as pathogenic or deleterious and were reviewed against the criteria defined by the American College of Medical Genetics.
The following variables were considered as critical for ranking the pathogenic variants: (a) coding regions; (b) rare variants ≤ 0.1% (0.001) MAF; (c) ultrarare variants ≤ 0.01% (0.0001) MAF; and (d) potentially novel variants in the public human population genome reference data sets with various ethnicities. The following reference datasets were used to estimate allele frequency: (a) Mixed control datasets (gnomAD); (b) disease data sets (Genotype to Mendelian Phenotype [Geno2MP v2.4]); (c) Indian and White Wellderly control data sets including healthy aging Indian exomes (www.instem.res.in/IndiCardiome) and the Wellderly data set (43); (d) South Asia–specific Indian control data sets including Genome Asia 100 K (44), IndiGenomes, and South Asian (Indian) healthy controls (4). The cardiac-specific expressing genes were obtained from the human protein atlas (45) and single cell genomics data (46) and include all the known cardiac development, physiology, and cardiomyopathy-associated genes. The presence of pathogenic variants in all cardiac-related genes were filtered and analyzed.

Generation of iPSCs. Patient-specific PBMCs were isolated using the density gradient method with Histopaque (MilliporeSigma, 1077). iPSCs were generated using the Cytotune2.0 Sendai virus kit (Invitrogen, A16517).
Initially, cells were seeded in a well of 24-well plate at a cell count of 3.5 × 105 per µL with 95% viability on day –4, in complete PBMC media. PBMC medium consisted of complete StemPro-34 medium (Invitrogen, 10639011) supplemented with the appropriate cytokines (SCF c-kit 100 ng/ mL [Invitrogen, PHC2115], FLT-3 100 ng/mL [Invitrogen, PHC9414], IL-3 20 ng/mL [Invitrogen, PHC0034], and IL-6 20 ng/mL [Invitrogen, PHC0064] at their final concentration). On day 0, cells were transduced with Sendai reprogramming vectors at appropriate MOIs. The volume of virus used was calculated using the following equation: V= (MOI [CIU/cell] × number of cells) / titer of virus (CIU/cell) × 10–3 (mL/µL), where V = volume of virus used for reprogramming as follows: Klf4 = (3 × [1 × 105]/1.1 × 10–3 × 10–5 = 2.7 µL; c-myc = (5 × [1 ×

105]/1 × 108 × 10–3 = 5 µL; and KOS(hKlf4,hOCT3/4 and hSox2) = (5 × [1 × 105]/9.8 × 107 × 10–3 =5.1 µL).

On day 1, the medium was replaced with PBMC medium to remove the viral titer. On day 3, cells were plated onto vitronectin-coated plates in complete StemPro-34 medium without cytokines. On day 7, transitioning began in Essential-8 medium (Thermo Fisher Scientific, A1517001). From day 8 to day 28, the medium was changed every day and was checked for iPSCs colony emergence. After stable proliferation of iPSCs colonies, cells were passaged and expanded for characterization and differentiation.

TTL p.G219S isogenic line generation using CRISPR/Cas9 gene editing. An isogeneic line was generated using CRISPR-Cas9 system by replacing the WT with the mutant nucleotide (c.655G>A) in a healthy control iPSC line. RNP complex was formed with 1mg Cas9 protein (Invitrogen, A36498), 5 mM sgRNA (Synthego, Target site: TAATATCTACCTCTATAGAG) and 5 mM ssODN (Single stranded oligoneucelotide) HDR template (IDT, Sequence-gcagAAGCTGGGTCTTGGTGGATCATCAGTATAATATCTACCTCTATAGAGA-GaGTGTGCTTCGGACTGCTTCAGAACCATATCATGTTGATAATTTCCAAGACAAAACCT-GCC) and electroporated in 100,000 cells using Neon transfection kit 10 mL (Invitrogen, MPK1025R) with the following pulse settings: 1150 V, 30 ms pulse width, 1 pulse. Two days after electroporation of cells, single-cell seeding was done in 96 wells, and cells were collected once appropriate colony size was reached. DNA was isolated using DNAzol (Invitrogen, 10503027), and target region was amplified using PCR. Purified PCR products were sent for sanger sequencing for mutation confirmation.

Embryoid body formation. Embryoid bodies are aggregates of ESCs or iPSCs that can form a 3D structure in a differentiated manner. Over the course of 2–4 days, all 3 germ layers ectoderm (outer layer), mesoderm (middle layer), and endoderm (inner layer) are present in differentiated embryoid bodies. For embryoid formation, after passaging iPSCs with ReleSR (Stem Cell Technologies, 100-0484) for 3 minutes, cells were removed by gentle pipetting into a 15 mL falcon tube containing 5 mL of iPSCs culture media, Stemflex (Thermo Fisher Scientific, A3349401), and pelleted at 200 rcf for 5 minutes. The supernatant was removed, the cell pellet was resuspended in 1 mL of Stemflex medium, and 10 µL of cells were taken out for cell counting. Cells were mixed with 10 µL of Trypan blue (Thermo Fisher Scientific, 15250061) and counted using a Countess 3 automated cell counter. Approximately 30,000 cells were seeded into each well of an ultra-low–attachment 24-well plate (Corning, CLS3473) with 500 µL of Stemflex media. Cells were then allowed to form aggregates for next 24–48 hours and then taken out for RNA isolation.

Germ layer marker analysis. Isolated RNA from fully formed embryoid bodies was converted to cDNA using Verso cDNA synthesis kit (Thermo Fisher Scientific, AB1453A) and the generated cDNA was subjected to PCR as a template against different germ layer markers like *GATA4* (mesoderm), *NODAL* (endoderm), *SOX1* and *NES* (ectoderm), and *RNU6-1* (housekeeping gene).

qPCR. Total RNA was extracted from iPSC-CMs. The cDNA was synthesized using 1 mg RNA and Verso cDNA synthesis kit. cDNA was amplified using real-time PCR. Data were analyzed using the 2ΔΔCT method. Relative fold changes in gene expression were normalized to *RNU6-1* or *GAPDH*. All the relevant primers used in the study are outlined in Supplemental Table 1.

Karyotyping. iPSCs generated at 50% confluency were karyotyped using the Giemsa (G-banding) cytogenetic staining technique to detect condensed chromosomes.

Characterization of pluripotency. Pluripotency status of the generated iPSCs were checked using immunocytochemical staining against different pluripotency markers like Oct3/4 (Santa Cruz Biotechnology Inc., sc-5279), SSEA3 (Santa Cruz Biotechnology Inc., sc-21703), SSEA4 (Santa Cruz Biotechnology Inc., sc-21704), Tra 1-60 (Santa Cruz Biotechnology Inc., sc-21705), and Tra 1-81 (Santa Cruz Biotechnology Inc., sc-21706). The protocol mentioned above for immunocytochemical staining was also followed in this experiment.

CM differentiation. Initially, patient-specific iPSCs and control line (WT) were acclimatized to mTeSr medium (STEMCELL Technologies, 100-0276), and cells were seeded in Matrigel-coated (Merck Corning, CLS354234) 12-well plates for differentiation. After reaching 70%–80% confluency, differentiation was started with induction by 4 μM CHIR99021 (Sigma-Aldrich, SML1046) for 48 hours followed by 5 μM IWP2 (Wnt inhibitor) (Sigma-Aldrich, I0536) for 2 days in cardiac differentiation medium (RPMI1640 with Glutamax [Thermo Fisher Scientific, 61870036], 213 μg/mL ascorbic acid [Sigma-Aldrich, A92902], 500 μg/mL human serum albumin [Sigma-Aldrich, A1887], and B27 minus insulin [Thermo Fisher Scientific, A1895601]). Cells were maintained until day 7 in cardiac differentiation media and were then replaced with cardiac maintenance media (RPMI1640 with Glutamax and B27 supplement [Thermo Fisher Scientific, 17504044]) for the next 10 days. On day 15 and 16, cells were transitioned to lactate medium (Sodium L-lactate [Sigma-Aldrich, 71718], RPMI1640 minus Glucose [Thermo Fisher Scientific, 11879020], 213 μg/mL ascorbic acid, and 500 μg/mL human serum albumin) for 4 days for purification of CMs. After recovery of cells in cardiac maintenance media for 4 days, cells were passaged and plated onto freshly coated Matrigel plates/dishes and with cardiac maintenance media (RPMI1640 with Glutamax, B27 supplement, and 5% knockout serum [Thermo Fisher Scientific, 10828028]) until day 30 or 40.

Immunocytochemical staining of iPSC-CMs. iPSC-CMs from the patient and the control line were dissociated and seeded on Matrigel-coated Ibidi confocal dishes (Ibidi, 81156). Cells on the dishes were washed 3 times with PBS (Thermo Fisher Scientific,10010023) and then fixed with 4% paraformaldehyde (Sigma-Aldrich, 158127) for 15 minutes at room temperature (RT). Again, coverslips were washed thoroughly with PBS 3 times and permeabilized afterward with 0.3% Triton-X100 (Sigma-Aldrich, 93443) for 15 minutes at RT. Cells were then blocked with 3% BSA (Himedia, MB083), 1% NGS (Himedia, RM10701), 0.3% Triton-X100 for 1 hour at RT. Primary antibody, ACTN2 (Invitrogen, MA1-22863, dilution 1:500), detyrosinated α-tubulin (Abcam, ab48389, dilution 1:500), desmin (Thermo Fisher Scientific, MA5-32068, dilution 1:500), NRF2 (Cell Signaling Technology, 12721, dilution 1:100), and cardiac troponin T (Thermo Fisher Scientific, MA5-12960, dilution 1:100) were added onto dishes overnight at 4°C

with respect to experiments requirements. The next day, dishes were washed 3 times with PBST (PBS+0.1% Tween 20 [Sigma Aldrich, P2287]) and then secondary antibody was added to the dishes (Alexa Fluor 488, 546, 647 [Thermo Fisher Scientific, A-11008, A-21244, A-11010, A-11001, A-21235], dilution 1:250) in accordance with the required experiment) for 1 hour at RT and then washed 3 times with PBST. After this, Phalloidin 546 (Thermo Fisher Scientific, A22283, dilution 1:400) along with Hoechst (Thermo Fisher Scientific, 62249, dilution 1:10,000) was added to the dishes for 1 hour at RT and then washed with PBST 3 times and imaged on an Olympus FV3000, 20×/60× NA 1.40 oil immersion objective.

Cell lysate preparation. Cells were lysed in RIPA buffer (Thermo Fisher Scientific, 89901) with 1× protease and phosphatase inhibitor cocktail (Thermo Fisher Scientific, A32959). Protein concentrations of cell lysates were measured using the BCA method, and approximately 15–20 μg of total protein was loaded onto gels and separated by SDS-PAGE.

Immunoblotting. Protein gels (SDS-PAGE) were transferred onto polyvinylidene difluoride (PVDF) membranes (Biorad, 1620177**)**. Membranes were blocked in 3% BSA and incubated with primary antibodies overnight at 4°C. Membranes were then incubated with appropriate secondary antibodies conjugated to horseradish peroxidase (Invitrogen, 31460 and 31431), and signal intensities were visualized by chemiluminescence.
The antibodies used are as follows: NRF2 (Cell Signaling Technology, 12721), SOD1 (Thermo Fisher Scientific, MA1105), NQO1 (Thermo Fisher Scientific, MA1-16672), GCLC (Thermo Fisher Scientific, MA5-26346), TTL (Thermo Fisher Scientific, PA5-66871), tyrosinated α-tubulin (Sigma-Aldrich, SAB4200776), detyrosinated α-tubulin (Abcam, ab48389), α-tubulin (Sigma-Aldrich, T9026), phospho-ERK1/2 (Cell Signaling Technology, 9101), ERK1/2 (Cell Signaling Technology, 9102), GAPDH (Thermo Fisher Scientific, MA5-15738), phospho-AKT (Cell Signaling Technology, 4060), AKT (Cell Signaling Technology, 4691), Desmin (Sigma-Aldrich, D1033), SERCA2 (Cell Signaling Technology, 9580), phospho-PLN (Cell Signaling Technology, 8496), and PLN (Cell Signaling Technology, 14562).

Purification of recombinant TTL proteins. Human TTL (hTTL) variants (WT and G219S) with a C-terminal 6XHis-tag were overexpressed in *E. coli* (bl21) cells and purified as described previously with some modifications. Transformed *E. coli* cells were induced with 0.5 mM IPTG (Sigma-Aldrich, I6758) overnight at 27°C to initiate protein expression. After induction, cells were harvested by centrifugation at 100,000*g* for 10 minutes at 4°C, and the resulting cell pellet was washed 2 times with PBS. The pellet was resuspended in cold lysis buffer and sonicated on ice for 10 cycles. The soluble cell lysate was purified using a His Trap column (cytivia, 17524802). The soluble fraction was equilibrated to a final concentration of 10 mM Imidazole (Sigma-Aldrich, I2399) before loading onto the column. The column was thoroughly washed using wash buffer, and the bound hTTL was eluted with elution buffer containing 500 mM Imidazole. Following elution, enriched hTTL fractions were pooled and further purified using a Sephadex200 16/60 column (Cytiva, 28989335) connected to an FPLC purification system. The purity and concentration of the protein were determined using SDS-PAGE.

Tubulin tyrosination assay. Tubulin was purified from the goat brain using 2 cycles of polymerization and depolymerization. CPA (Sigma-Aldrich, C9268) treatment of purified goat brain tubulin was performed. A reaction mixture containing purified goat brain tubulin with 20% glycerol, 2 mM GTP, and 2.5 μg/mL of pancreatic CPA was prepared in 1× BRB80 buffer (80 mM Pipes [Sigma-Aldrich, P6757], 1 mM $MgCl_2$ [Sigma-Aldrich, 7786-30-3], and 1 mM EGTA, [Sigma-Aldrich, E3889] pH 6.8, with KOH). Briefly, the mixture was incubated on ice for 10 minutes, and the reaction was initiated by incubation at 37°C for 20 minutes. After incubation, the reaction was immediately stopped by adding 20 mM DTT (Sigma-Aldrich, D9779), and the MTs were further allowed to polymerize for 10 minutes at 37°C. Polymerized MTs were then pelleted at 100,000*g* for 40 minutes at 37°C and were further resuspended in ice-cold BRB80. A reaction mixture (10 μL) containing CPA-treated tubulin (2.5 μM) in 50 mM MES/K, 100 mM KCl, 10 mM MgCl2, 5 mM DTT, 2.5 mM ATP, and 0.2 mm L-tyrosine was used to analyze the tyrosination abilities of TTL variants. The mixture was incubated with TTL at a molar ratio of 1:0.0002 for different time points (0, 5, 10, 15, 20, and 30 minutes) at 37°C in a circulating water bath. The enzymatic reaction was stopped using 2× SDS-PAGE Sample Buffer. The resulting samples were subsequently analyzed using Western blotting and were probed using tyrosinated α-tubulin (Sigma-Aldrich; clone YL1/2, MAB1864-I) (1:5,000) and α-tubulin (Sigma-Aldrich, T9026) (1:5,000), followed by anti–mouse or –rabbit peroxidase–conjugated secondary antibody (1:5,000). Image J was used to quantify the relative intensities of the Western blot bands.

Global RNA-Seq and analysis. RNA was isolated from the respective CMs using the Trizol (Thermo Fisher Scientific, 15596026) RNA extraction method. Sequencing was performed at the institutional sequencing facility. RNA integrity was checked using a Bioanalyzer, and samples with RNA integrity number (RIN) above 9 were used for analysis. cDNA was prepared according to the manufacturer's instructions using the NEBNext Ultra Directional RNA Library Prep Kit for Illumina (NEB, E7760). For mRNA enrichment, the polyA selection method was employed. Next-generation sequencing of libraries was performed on the Illumina HiSeq 2500 platform for 1 × 100 bp at ~30 million to 35 million reads per sample.

Sequencing data were trimmed for adaptor sequences and low-quality reads (~15 bp) using Trim Galore! and mapped to GRCh38 using Top Hat. Paired-end reads obtained from both ends of the same RNA fragment were examined, and paired reads ≥ 35 bp were retained. DEGs between the samples were identified using Cufflinks/Cuffdiff software with parameters: Log_2 fold change ± 1.0 and adjusted $P < 0.05$. Gene ontology of biological processes (using EnrichR) was used to show the network of significantly enriched biological processes and pathways.

ROS measurement and analysis. Before staining, the CMs were seeded on suitable culture dishes and allowed to adhere and grow into the ideal morphology. To measure ROS, DHE (Sigma-Aldrich, 309800) was employed. DHE powder was dissolved in dimethyl sulfoxide (DMSO [Sigma Aldrich, D2650]) to form a stock solution at a concentration of 5 mM. To obtain a working concentration of 5 μM, the stock solution was diluted in PBS. The cells were initially rinsed with PBS and then exposed to DHE staining solution at 37°C in the dark for 30 minutes. This incubation period allowed DHE to penetrate cells and undergo oxidation by ROS.

After the incubation period for staining, the staining solution was carefully pipetted out, and the cells were gently rinsed with PBS to eliminate any surplus dye. The cells were subsequently

treated with 4% paraformaldehyde solution for 5 minutes at RT to maintain the integrity of the staining pattern. The stained cells were imaged using a confocal microscope that had the necessary filter sets for DHE, with excitation/emission wavelengths of 488/610 nm. Optimal exposure settings were employed to capture a balanced range of fluorescence intensities, avoiding overexposure. These parameters were consistently maintained for all samples.

Statistics. Depending on the experiments, statistical significance was determined by 2-tailed Student's *t* test, 2- tailed multiple *t* test, and 1-way ANOVA with post hoc Tukey's test. $P < 0.05$ was considered to be statistically significant.
