# Supplementary material for: Tubulin tyrosine ligase variant perturbs microtubule tyrosination, causing hypertrophy in patient-specific and CRISPR gene-edited iPSC-cardiomyocytes
Source: JCI Insight. 2025 Aug 8;10(15):e187942. doi: 10.1172/jci.insight.187942 (PMC12333943; doi:10.1172/jci.insight.187942)

Figure 1D

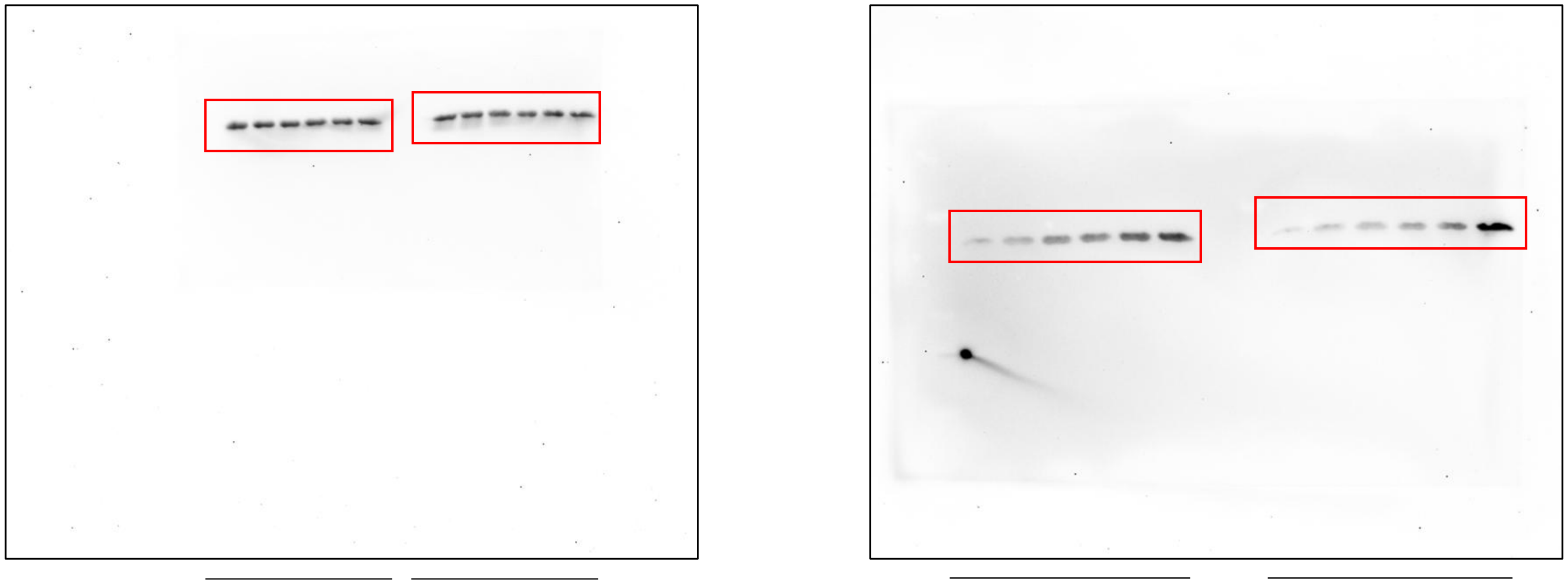

Figure 3C

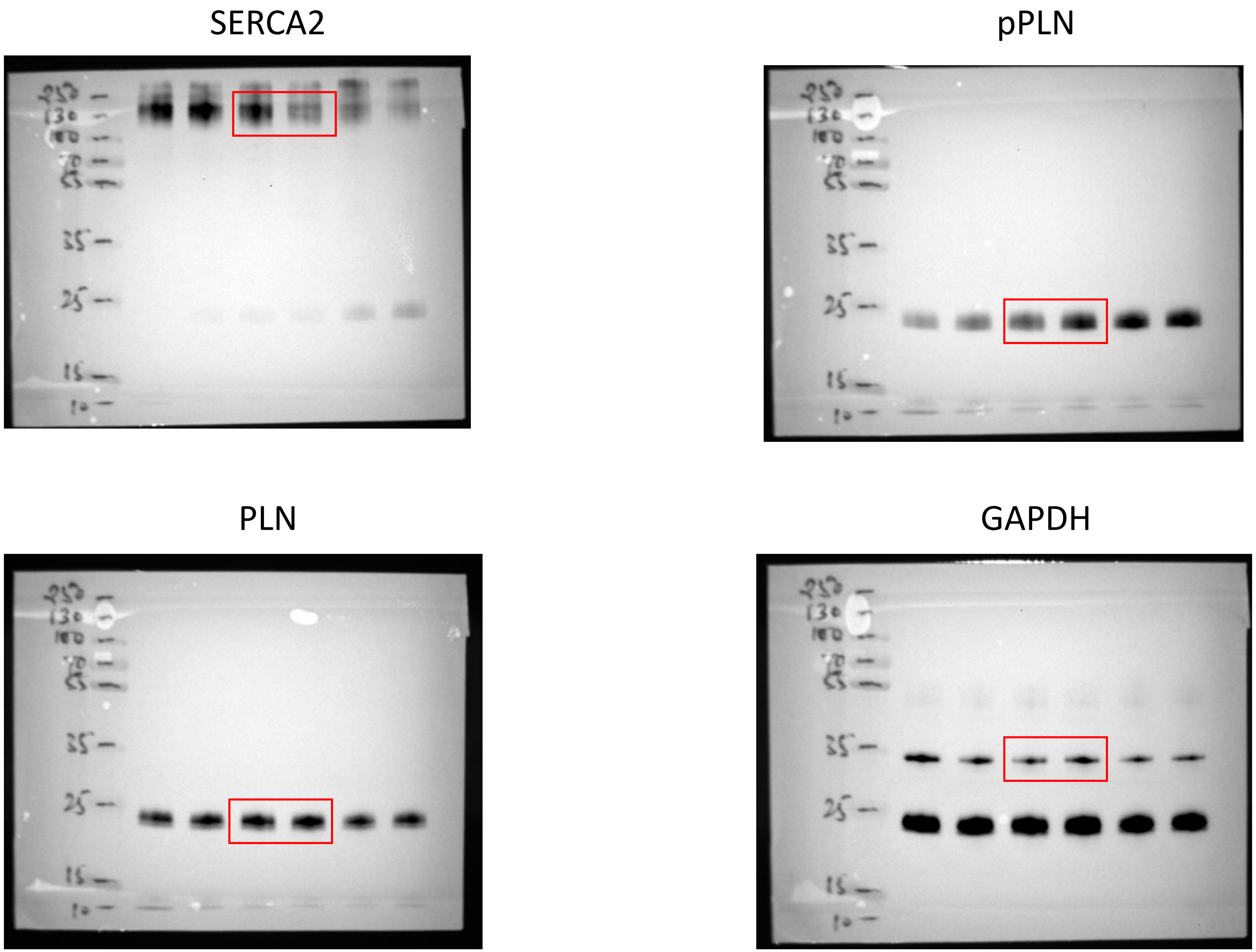

Figure 4C

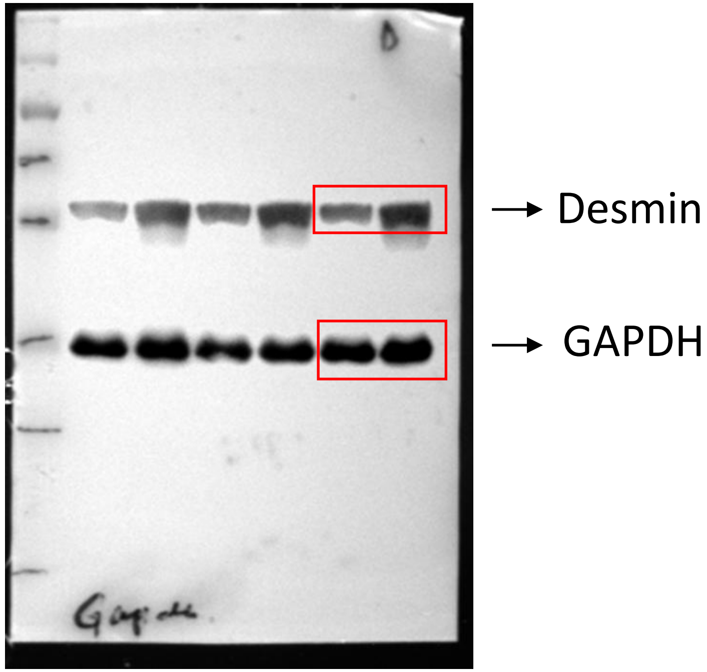

Figure 4D

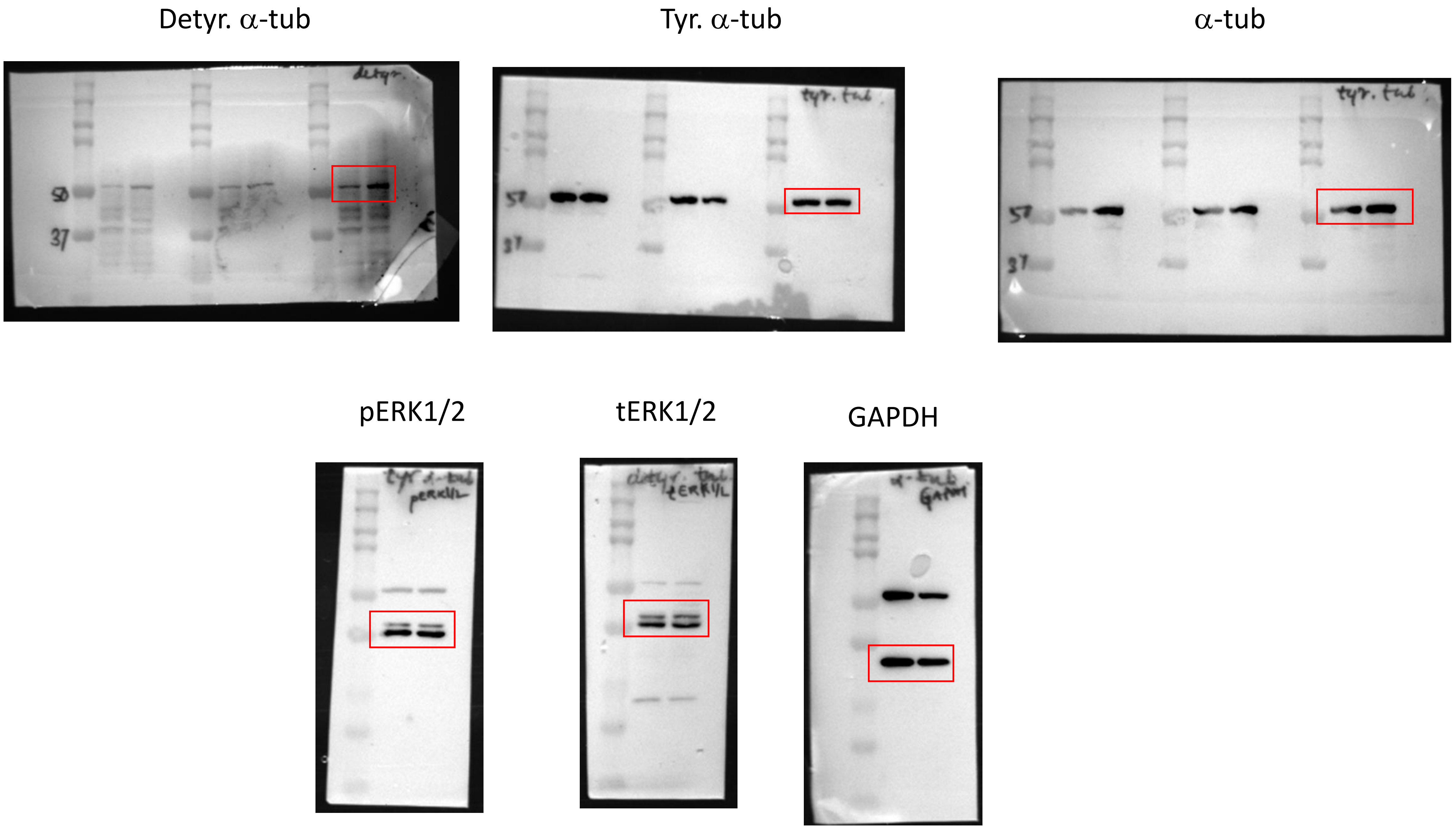

Figure 5D

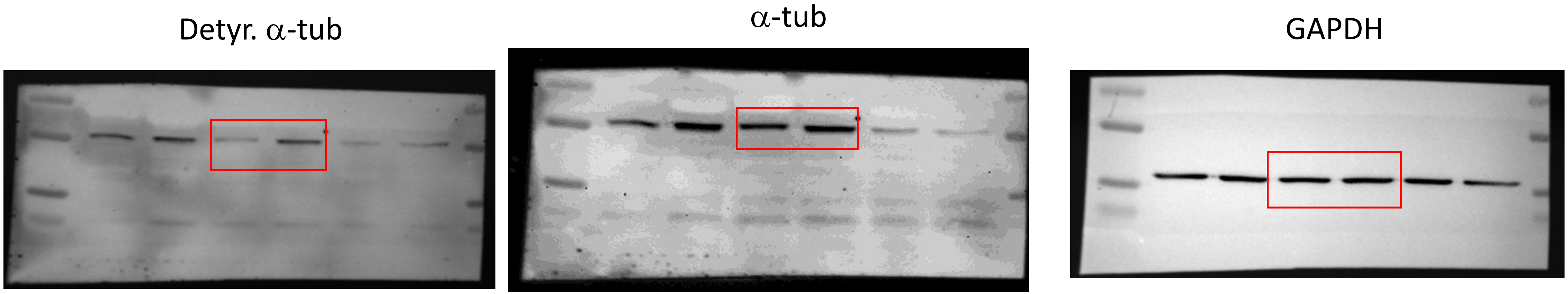

Figure 6E

NRF2 pAKT tAKT GAPDH

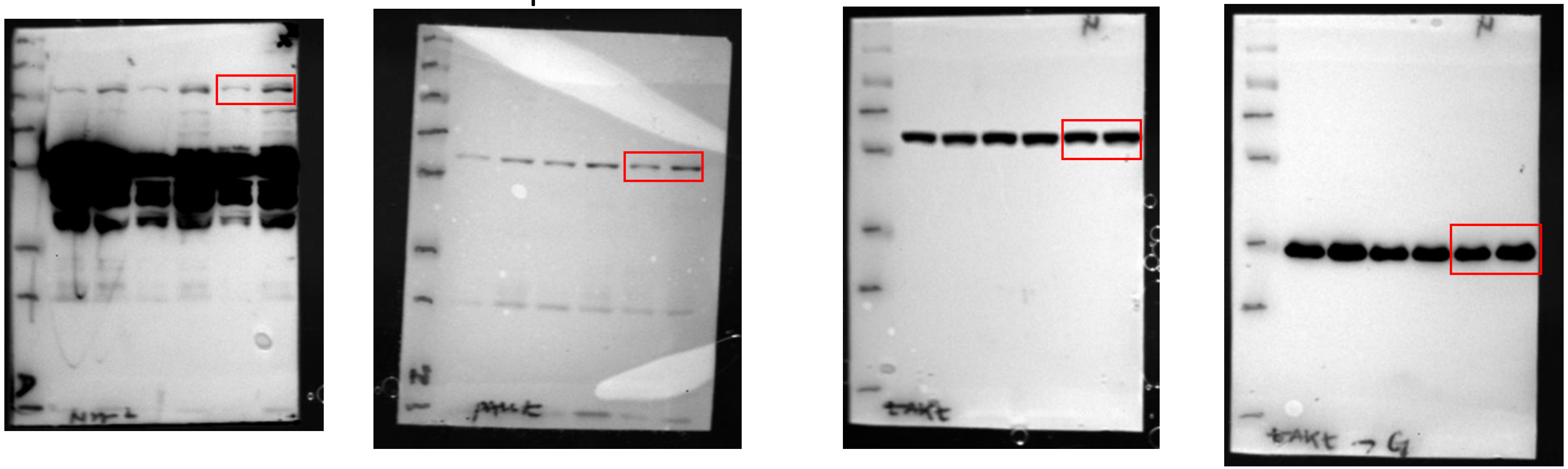

Figure 6H

NRF2

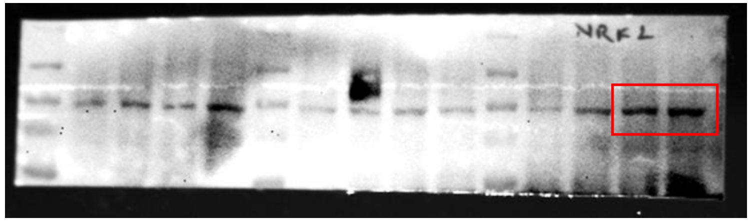

GCLC

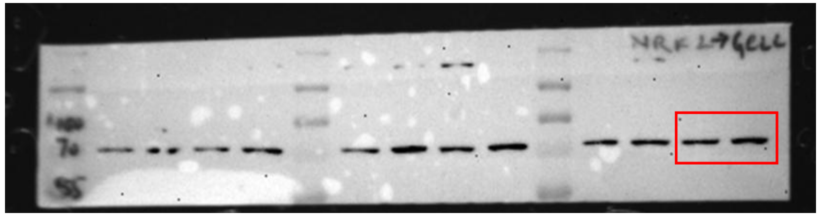

GAPDH

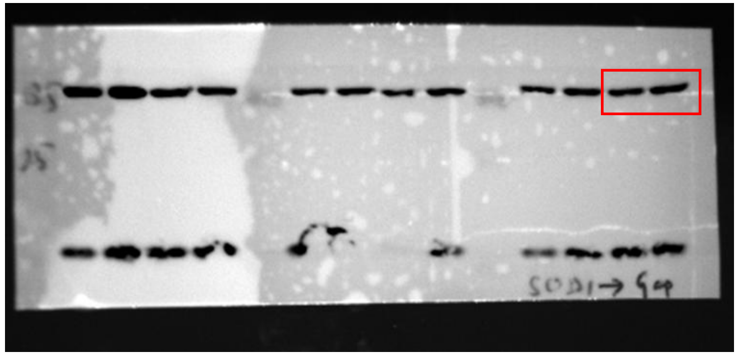

SOD1

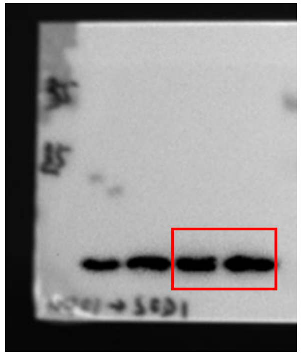

NQO1 membrane

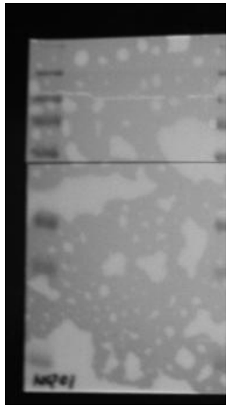

NQO1 chemi

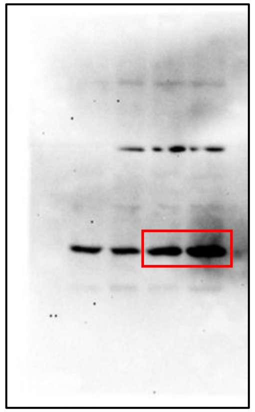

Figure 7C

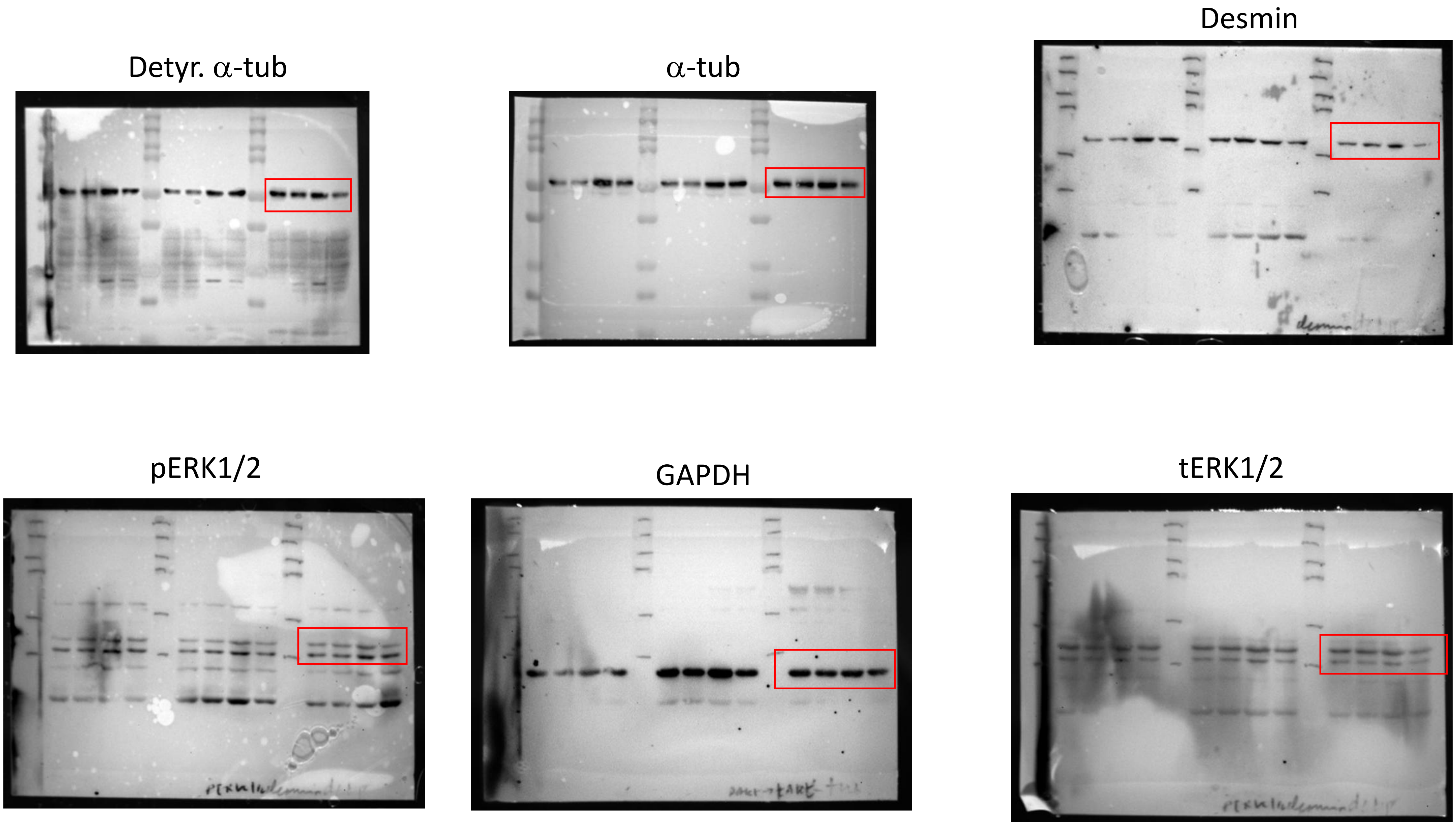

Figure 8B

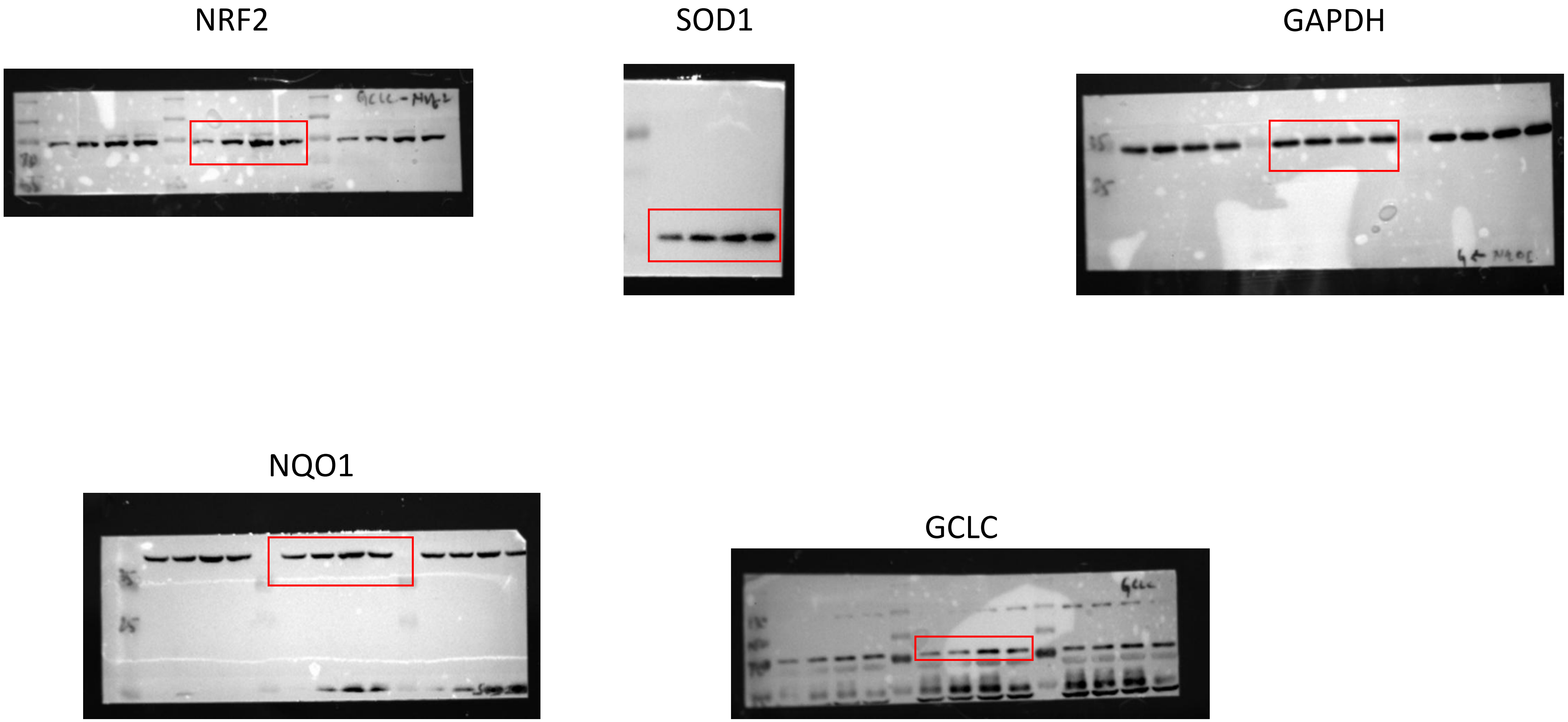

Supplement: Unedited blot and gel images [file jciinsight-10-187942-s165.pdf]
